# Novel 6-Aminoquinazolinone Derivatives as Potential Cross GT1-4 HCV NS5B Inhibitors

**DOI:** 10.3390/v14122767

**Published:** 2022-12-12

**Authors:** Tamer Nasr, Ahmed M. Aboshanab, George Mpekoulis, Antonios Drakopoulos, Niki Vassilaki, Grigoris Zoidis, Khaled A. M. Abouzid, Wafaa Zaghary

**Affiliations:** 1 Department of Pharmaceutical Chemistry, Faculty of Pharmacy, Helwan University, Ain-Helwan, Cairo 11795, Egypt; 2 Department of Pharmaceutical Chemistry, Faculty of Pharmacy, MTI University, Cairo 12055, Egypt; 3 Molecular Virology Laboratory, Hellenic Pasteur Institute, 11521 Athens, Greece; 4 Department of Chemistry and Molecular Biology, University of Gothenburg, SE-412 96 Gothenburg, Sweden; 5 Department of Pharmacy, Division of Pharmaceutical Chemistry, School of Health Sciences, National and Kapodistrian University of Athens, Panepistimiopolis Zografou, 15771 Athens, Greece; 6 Department of Pharmaceutical Chemistry, Faculty of Pharmacy, Ain Shams University, Abbassia, Cairo 11566, Egypt

**Keywords:** 6-Aminoquinazolinone, molecular docking, Hepatitis C, Anti HCV, NS5B inhibitors, daclatasvir

## Abstract

Chronic hepatitis C virus (HCV) infections are a worldwide medical problem responsible for diverse types of liver diseases. The NS5B polymerase enzyme has become a very interesting target for the development of anti-HCV drugs owing to its fundamental role in viral replication. Here we report the synthesis of a novel series of 1-substituted phenyl-4(1*H*)-quinazolinone and 2-methyl-1-substituted phenyl-4(1*H*)-quinazolinone derivatives and evaluate their activity against HCV in HCV subgenomic replicon assays. The biological data revealed that compound **11a** showed the highest activity against HCV GT1b at a micromolar concentration (EC_50_ = 0.984 µM) followed by compound **11b** (EC_50_ = 1.38 µM). Both compounds **11a** and **11b** had high selectivity indices (SI = CC_50_/EC_50_), 160.71 and 71.75, respectively, which make them very interesting candidates for further development of more potent and selective anti-HCV agents.

## 1. Introduction

HCV is the main causative agent of chronic hepatitis [1,2,3,4,5]. Current HCV treatment protocols focus on using combinations of two or more direct-acting antiviral drugs (DAAs) [6,7,8,9,10,11,12,13,14,15,16,17]. Within the host cell, the single polyprotein produced after the viral RNA translation is cleaved into structural proteins and nonstructural proteins (NS3/4A, NS4B, NS5A, and NS5B), which are involved in viral replication and production [18,19,20]. NS5B, an RNA-dependent RNA polymerase (RdRp), plays a very important role in viral synthesis and replication [21,22]. The lack of a functional counterpart in mammalian cells made it a very valuable target for the development of selective and non-toxic anti-HCV drugs [23]. Similar to other RNA polymerase enzymes, HCV NS5B reveals a characteristic right-hand topology with palm, finger, and thumb domains [24,25,26,27]. Regarding these structural patterns, NS5B inhibitors are divided into two categories: Nucleoside inhibitors, NIs (Sofosbuvir), which act as false substrates for the enzyme, thus blocking the elongation of the RNA chain; and nonnucleoside inhibitors, NNIs (Beclabuvir, filibuvir, lomibuvir, Dasabuvir, setrobuvir, and tegobuvir), which change the conformation of the active site and stop the initiation of RNA replication processes upon binding to the different enzyme allosteric sites [20,27,28,29,30]. Of these binding allosteric sites, the thumb pocket II (TP-2) binding site has been intensely investigated in the last decade [31]. Crystal structures of NNIs bound to this TP-2 binding site have been published [32,33,34,35,36].

**Figure 1:**
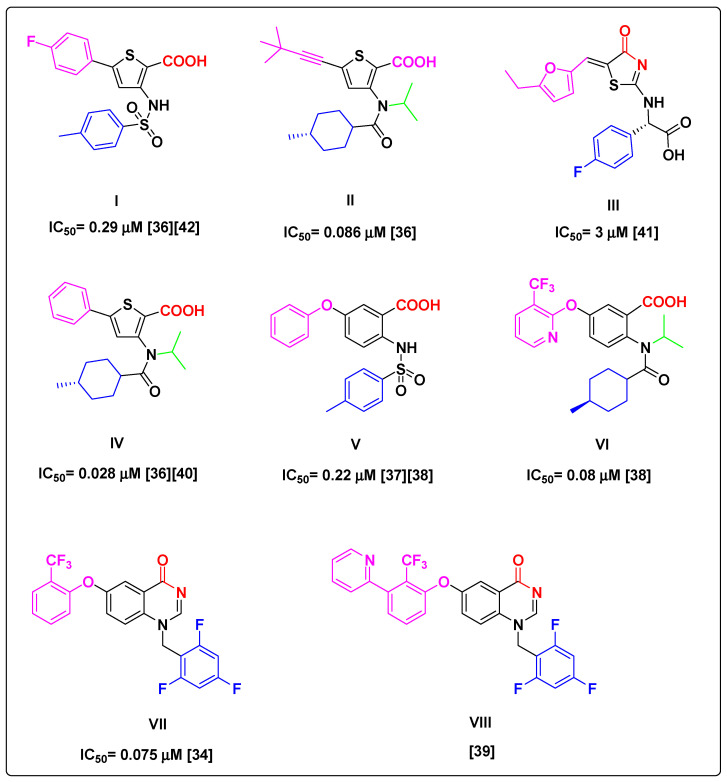
Some reported NS5B TP-2 inhibitors exhibiting promising HCV inhibition. The crystal structure of compounds I, II, III, IV, V, VII, and VIII bound to TP-2 binding site of NS5B were reported; PDB codes: 2D3Z, 4EO6, 2HWI, 2GIR, 4J08, 4JTW, and 4JU2, respectively.

Studying these crystal structures revealed that most of the above NNIs have the same conformational patterns and binding interactions with the NS5B TP-2 allosteric site. Two direct or water-mediated hydrogen bonds between the inhibitor molecules and backbone NHs of Ser476 and Tyr477 were observed. Furthermore, the inhibitor’s hydrophobic moieties bind with two hydrophobic pockets of the enzyme via stacking with the side chain of Leu419, Met423, Leu497, and Trp528 [34,35,36]. Recently reported NS5B TP-2 inhibitors possessing micromolar inhibition potency in both enzymatic and cell-based replicon assays are depicted in Figure 1 and Table 1, respectively [34,36,37,38,39,40,41,42]. 

All lead structures share similar motif features and properties required for successful binding to the TP-2 allosteric site despite bearing different pharmacophoric elements. Specifically, motifs in red provide a dual HB formation between substrates and protein residues Ser476/Tyr477 (Figure 1). These key interactions are reported to be satisfied equally by the carboxylate (i.e., compounds **I**, **II**, **IV**, **V,** and **VI**), thiazolone (i.e., compound **III**), and quinazolinone (i.e., compound **VII** and **VIII**) moieties. Aryl substituents in blue (Figure 1) are well occupied in a deep hydrophobic pocket stacking with the side chain of Leu419, Arg422, and Trp528. The *N*-isopropyl group in green (Figure 1) in compounds **II**, **IV,** and **VI** points to the solvent and keeps the orientation of the cyclohexyl amide moiety toward the deep lipophilic pocket. In the rest of the molecules, the left-hand-side moieties in pink also make contact with the left-hand hydrophobic pocket of TP-2, stacking with the side chain of Leu497 and Ilu482. However, these lead compounds exhibit many drawbacks. Compound **III** has low potency in enzymatic assays (IC_50_ = 3 μm) [41]. Furthermore, compound **V** showed no cellular potency during an HCV subgenomic replicon assay (EC_50_ > 30 μm) [37]. Although compounds **I**, **II**, **VI, VII,** and **VIII** exhibited potent in vitro anti-HCV activity in both enzymatic and subgenomic cell replicon assays, they showed high lipophilic character (Table 1). Moreover, even though NS5B polymerase inhibitors exhibit broad activity against HCV genotypes, they have a low genetic barrier to resistance [29,43,44].

**Figure 2:**
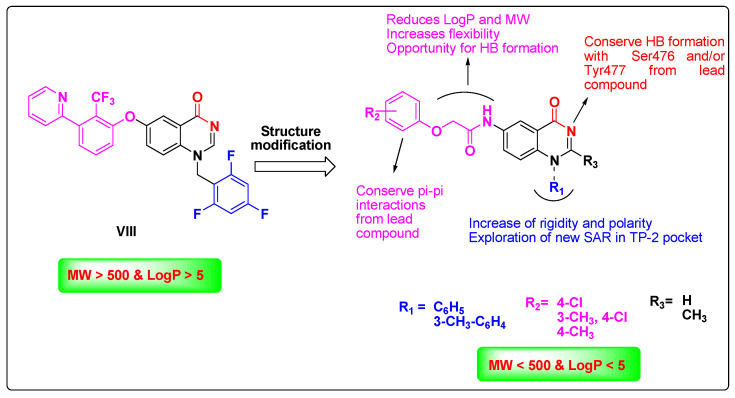
Rationale of the designed target compounds to act as NS5B inhibitors that fulfil Lipinski’s rule of five.

Based on the aforementioned information, we became interested in designing new potent NNIs against NS5B, able to bind the TP-2 allosteric site while fulfilling Lipinski’s rule of five. Thus, in the present study, we report the synthesis and biological evaluation of a novel series of 1-substituted phenyl-4(1*H*)-quinazolinone **7a**–**f** and 2-methyl-1-aryl substituted-4(1*H*)-quinazolinone derivatives **11a**–**f** (Figure 2), which maintain the important structural features of the lead compound **VIII**, but with ClogP value less than 5, molecular weights less than 500, and improved water solubility properties (LogS). Most importantly, the amide moiety of the quinazolinone ring was conserved in order to form the essential hydrogen bonds with the protein residues Ser476 and Tyr477 (Figure 2). Nonetheless, the challenge in our ligand design was whether the phenylquinazolinone scaffold could enter and reside in the hydrophobic TP-2 pocket of the benzylquinazolinone analogues’ binding mode. Although the volume of the TP-2 pocket is low as shown in the respective crystal structures, this part consists of flexible side chains, which could move aside, given the rigidity of the scaffold. Thus, there would be a potential of discovering new SAR, valuable for the drug design of this biomolecular target. The four-atom linker was designed to reduce both the molecular weight and ClogP values of the target compounds. R1 was carefully selected as phenyl or m-tolyl to study their potential stacking in the deep hydrophobic pocket of the enzyme and to increase the polarity of the compound. Although these groups increase the rigidity of the whole molecule, prospective SAR in this specific region of the TP-2 pocket has not been explored. The C-2 methyl group was placed as a means of increasing the ligand volume near the phenyl ring, in an effort to facilitate the approachability of the phenyl ring at N1 in the deep hydrophobic pocket by making it more accessible. Finally, R2 was carefully selected to yield the *π*–*π* interactions with the second hydrophobic pocket of the enzyme. Finally, in this study, we also evaluate the antiviral activity of the target compounds against different HCV genotypes.

## 2. Materials and Methods

### 2.1. Chemistry

Starting materials, solvents, and reagents were purchased from Sigma Aldrich (St. Louis, MO, USA), Acros Organics (Thermo Fisher Scientific, Bridgewater, NJ, USA), and Alfa Aesar (Kandel, Germany). Melting points were determined on a Stuart melting point apparatus (Stuart Scientific, Redhill, UK) using open capillary tubes and were uncorrected. Analytical thin-layer chromatography (TLC) was employed routinely to monitor the progress of the reactions and check the purity of the products using thin layer (0.2 mm) aluminum sheets (20 × 20 cm) coated with silica gel F254 (Merck, Darmstadt, Germany). Spots were visualized using a UV lamp at λmax 254 nm. The eluent was a hexane/ethyl acetate mixture (2:1). Column chromatography was performed using silica gel (pore size 60 Å, 230–400 mesh particle size, Merck, Darmstadt, Germany); elution was conducted using a hexane/ethyl acetate mixture (2:1). All evaporations were performed under reduced pressure at 40 °C. IR spectra (KBr disk) were recorded on an FT-IR spectrometer (Perkin Elmer, Waltham, MA, USA). NMR spectra were obtained using a Bruker NMR spectrometer (Bruker Biospin GmbH, Rheinstetten, Germany) operating at 400 MHz for ^1^H NMR and 100 MHz for ^13^C NMR at the Center for Drug Discovery Research and Development, Faculty of Pharmacy, Ain Shams University. Chemical shifts are expressed in δ values (ppm) relative to TMS as an internal standard using DMSO-*d_6_* as solvent. Elemental analyses were obtained using the Thermo Scientific Flash 2000 elemental analyzer (Thermo Fisher Scientific Inc., Waltham, MA, USA) at the Regional Center for Mycology and Biotechnology, Al-Azhar University. Electrospray ionization mass (ESI-MS) was obtained using a Thermo Scientific mass spectrometer LC-MS (Thermo Scientific Inc., Waltham, MA, USA) at the Center for Drug Discovery Research and Development, Faculty of Pharmacy, Ain Shams University. The analytical purity of target compounds was determined by reverse-phase HPLC in conjunction with product analysis by ESI-MS. UV absorption was detected from 200 to 800 nm using a diode array detector. Elution was achieved on a UPLC RP-C18 column (150mm × 2 mm; 2.7 µm) maintained at an ambient temperature with 70% acetonitrile in water as a mobile phase. The purity of the compounds was determined at 254 nm, and we observed a pure peak for each compound attributed to its mass. The purity of the compounds was found to be more than 95%. Compounds 5-nitro-2-(phenylamino)-benzoic acid **1a** [48], 5-nitro-2-(*m*-tolylamino)benzoic acid **1b** [49], and 5-nitro-2-(phenylamino)benzamide **3a** [50,51] were synthesized according to the reported procedures.

**General procedure for the synthesis of 5-nitro-2-(substituted phenyla-mino)benzamide derivatives (3a,b).**

A mixture of 5-nitro-2-(phenylamino)-benzoic acid **1a** (2 g, 7.75 mmol) or 5-nitro-2-(*m*-tolylamino)benzoic acid **1b** (2 g, 7.35 mmol) and phosphorus pentachloride PCl_5_ (8 g, 38.75 mmol) in methylene chloride (20 mL) was refluxed for 6 h and monitored by TLC until the reaction was complete. The crude solution of 5-nitro-2-(phenylamino)benzoyl chloride **2a** or 5-nitro-2-(*m*-tolylamino)benzoyl chloride **2b** in methylene chloride without separation was added dropwise to 25% aq ammonium hydroxide (20 mL). The reaction was stirred at room temperature for one hour, and the reaction was monitored by TLC until completed. Then the solvents were removed under a vacuum, and ice water (100 mL) was added to the residue to precipitate the target compounds. The precipitate was filtrated off, washed with water, and recrystallized from ethyl acetate affording the desired products.

**5-Nitro-2-(*m*-tolylamino) benzamide (3b).**

Yellow powder, yield 80%, mp 188–190 °C; IR (KBr) νmax/cm^−1^: 3437, 3344, 3194 (NH_2_ and NH), 3089 (CH-Ar), 2916 (CH-sp^3^), 1685 (C=O), 1539, 1330 (NO_2_); ^1^H NMR (400 MHz, DMSO-*d_6_*) δ 2.31 (s, 3H, CH_3_), 7.17–7.33 (m, 4H, Ar-H), 7.43 (d, *J* = 12 Hz, 1H, Ar-H), 7.75 (brs, 1H, NH_2_, exchangeable by D_2_O), 8.12 (d, *J* = 8 Hz, 1H, Ar-H), 8.51 (brs, 1H, NH_2_, exchangeable by D_2_O), 8.67 (s, 1H, Ar-H), 10.89 (brs, 1H, NH, exchangeable by D_2_O); ^13^C NMR (100 MHz, DMSO) δ 20.0 (CH_3_), 113.7, 115.1, 120.4, 122.3, 123.9, 125.8, 128.5, 130.3, 137.4, 138.8, 139.8, 151.5, 170.4; C_14_H_13_N_3_O_3_; LC-ESI-MS (*m*/*z*): 272.1 [M+H]^+^; purity was confirmed by HPLC-UV (254 nm)-ESI-MS.

**General procedure for the synthesis of 6-nitro-1-(substitutedphenyl)quinazolin-4(1*H*)-one (4a, 4b).**

To a stirring mixture of compound **3a** or **3b** (5 mmol) and triethyl orthoformate (10 mL) in ice path, sulfuric acid (2.5 mL) was added dropwise over 15 min. Then, the mixture was refluxed for 8 h (monitored by TLC). After the reaction’s completion, it was poured into ice water. The product was separated. The precipitated solid was filtrated off, washed with hot hexane, and dried in vacuo.

**6-Nitro-1-phenylquinazolin-4(1*H*)-one (4a).**

Greenish yellow powder, yield 85%, mp 246–248 °C; IR (KBr) νmax/cm^−1^: 3059 (CH-Ar), 2989 (CH-sp^3^), 1699 (C=O), 1550, 1334 (NO_2_); ^1^H NMR (400 MHz, DMSO-*d_6_*): δppm = 7.11 (d, 1H*, J* = 6 Hz, Ar-H), 7.71–7.79 (m, 5H, Ar-H), 8.48 (dd, 1H, *J* = 9.2 Hz and 4.8 Hz, Ar-H), 8.66 (s, 1H, Ar-H), 8.82 (d, 1H, *J* = 4 Hz, Ar-H); ^13^C NMR (100 MHz, DMSO) δ 108.1, 119.3, 119.6, 123.7, 128.3 (2C), 128.7, 130.9, 131.0 (2C), 137.8, 145.1, 155.3, 167.9 (CO); MS *m*/*z* (%): 267.08 (M+, 74), 77 (100); Anal. Calcd. for C_14_H_9_N_3_O_3_ (267.08): C, 62.92%; H, 3.39%; N, 15.72%; Found: C, 63.15%; H, 3.61%; N, 15.92%.

**6-Nitro-1-(*m*-tolyl)quinazolin-4(1*H*)-one (4b).**

Green powder, yield 85%, mp 185 °C; IR (KBr) νmax/cm^−1^: 3055 (CH-Ar), 2989 (CH-sp^3^), 1670 (C=O), 1527, 1334 (NO_2_); ^1^H NMR (400 MHz, DMSO-*d_6_*): δppm = 2.44 (s, 3H, CH_3_), 7.14 (d, 1H, *J* = 6 Hz, Ar-H), 7.51–7.79 (m, 4H, Ar-H), 8.48 (d, 1H, *J* = 12 Hz, Ar-H), 8.64 (s, 1H, Ar-H), 8.81 (s, 1H, Ar-H); ^13^C NMR (100 MHz, DMSO) δ 21.3 (CH_3_), 119.4, 119.50, 123.7, 125.3, 128.6, 128.7, 130.7, 131.2, 137.7, 140.8, 145.0, 145.1, 155.3, 167.9 (CO); C_15_H_11_N_3_O_3_; LC-ESI-MS (*m*/*z*): 282 [M+H]^+^; purity was confirmed by HPLC-UV (254 nm)-ESI-MS.

**General procedure for the synthesis of 6-amino-1-(substituted phenyl)quinazolin-4(1*H*)-one (5a, 5b).**

A mixture of compound **4a** or **4b** (5 mmol), stannous chloride dihydrate (5.6 g, 25 mmol), and conc. HCl (5 mL) in 15 mL ethyl acetate was stirred at room temperature for 2 h. The reaction was monitored with TLC. After the reaction was complete, the reaction mixture was diluted with a mixture of ethyl acetate and methanol (3:1) (200 mL) and the pH was adjusted to 7 through the addition of sodium carbonate powder. The solution mixture was filtered, and the organic filtrate was dried over sodium sulphate. Filtration of sodium sulphate was carried out, and the filtrate was evaporated under a vacuum to afford the crude products. The obtained residue was purified by silica gel chromatography using a hexane/ethyl acetate mixture (2:1) to give the desired compounds.

**6-Amino-1-phenylquinazolin-4(1*H*)-one (5a).**

White powder, 67%, mp 293–295 °C; IR (KBr) νmax/cm^−1^: 3425, 3336 (NH_2_), 3055 (CH-Ar), 2885 (CH-sp^3^), 1635 (C=O); ^1^H NMR (400 MHz, DMSO-*d_6_*) δ 5.61 (s, 2H, NH_2_, exchangeable by D_2_O), 6.66 (d, *J* = 6.4 Hz, 1H, Ar-H), 7.00 (d, *J* = 12 Hz, 1H, Ar-H), 7.26 (s, 1H, Ar-H), 7.55–7.65 (m, 5H, Ar-H), 8.24 (s, 1H, Ar-H); ^13^C NMR (100 MHz, DMSO) δ 108.1, 118.2, 121.3, 122.2, 128.0 (2C), 130.4, 130.7 (2C), 131.9, 138.1, 148.2, 151.0, 169.0 (CO); C_14_H_11_N_3_O; LC-ESI-MS (*m*/*z*): 238 [M+H]^+^; purity was confirmed by HPLC-UV (254 nm)-ESI-MS.

**6-Amino-1-(*m*-tolyl)quinazolin-4(1*H*)-one (5b).**

White powder, 65%, mp 288–290 °C; IR (KBr) νmax/cm^−1^: 3425, 3336 (NH_2_), 3059 (CH-Ar), 2981 (CH-sp^3^), 1631 (C=O); ^1^H NMR (400 MHz, DMSO-*d_6_*) δ 2.41 (s, 3H, CH_3_), 5.60 (s, 2H, NH_2_, exchangeable by D_2_O), 6.67–6.72 (dd, *J* = 11.2 Hz and 9.6 Hz, 1H, Ar-H), 6.98 (d*, J* = 8 Hz, 1H, Ar-H), 7.62 (s, 1H, Ar-H), 7.23– 7.68 (m, 4H, Ar-H), 8.22 (d, *J* = 7.2 Hz, 1H, Ar-H); ^13^C NMR (100 MHz, DMSO) δ 21.2, 108.0, 118.2, 121.3, 122.1, 127.3, 128.3, 130.4, 130.8, 130.9, 131.9, 138.1, 148.2, 150.9, 169.0(CO); C_15_H_13_N_3_O; LC-ESI-MS (*m*/*z*): 252 [M+H]^+^; purity was confirmed by HPLC-UV (254 nm)-ESI-MS.

**General procedure for the synthesis of 2-chloro-*N*-(4-oxo-1-substituted phenyl-1,4-dihydroquinazolin-6-yl)acetamide (6a, 6b).**

Chloroacetyl chloride (0.5 mL, 6.3 mmol) was slowly added to a magnetically stirred mixture of compound **5a** or **5b** (4.2 mmol) and K_2_CO_3_ (0.873 g, 6.3 mmol) in CH_2_Cl_2_ (10 mL). The reaction mixture was stirred at room temperature and monitored by TLC. After the reaction was complete, the solvents were evaporated, and ice water (100 mL) was added to the residue. The precipitate was filtered, washed with water, and left to dry in vacuo.

**2-Chloro-*N*-(4-oxo-1-phenyl-1,4-dihydroquinazolin-6-yl)acetamide (6a).**

Reddish white powder, 82%, mp < 300 °C; IR (KBr) νmax/cm^−1^: 3275 (NH), 3070 (CH-Ar), 2958 (CH-sp^3^), 1689 (C=O); ^1^H NMR (400 MHz, DMSO-*d*_6_) δ 4.28 (s, 2H, CH_2_), 6.94 (d, *J* = 8 Hz, Ar-H), 7.62–7.70 (m, 5H, Ar-H), 7.84 (d, *J* = 8 Hz, 1H, Ar-H), 8.44–8.45 (m, 2H, Ar-H), 10.69 (s, 1H, NH, exchangeable by D_2_O); ^13^C NMR (100 MHz, DMSO) δ 43.8, 116.8, 118.3, 120.3, 126.5, 128.1 (2C), 130.6 (2C), 130.8, 137.1, 137.2, 137.7, 153.0, 165.6 (CO); C_16_H_12_ClN_3_O_2_; LC-ESI-MS (*m*/*z*): 314 [M+H]^+^; purity was confirmed by HPLC-UV (254 nm)-ESI-MS.

**2-Chloro-*N*-(4-oxo-1-(*m*-tolyl)-1,4-dihydroquinazolin-6-yl)acetamide (6b).**

Reddish white powder, 84%, mp 290 °C; IR (KBr) νmax/cm^−1^: 3294 (NH), 3070 (CH-Ar), 2954 (CH-sp^3^), 1693 (C=O); ^1^H NMR (400 MHz, DMSO-*d*_6_) δ 2.40 (s, 3H, CH_3_), 4.31 (s, 2H, CH_2_), 6.94–7.00 (m, 1H, Ar-H), 7.38–7.74 (m, 4H, Ar-H), 7.86 (d, *J* = 12 Hz, 1H, Ar-H), 8.42 (s, 1H, Ar-H), 8.47 (s, 1H, Ar-H), 10.85 (s, 1H, NH, exchangeable by D_2_O); ^13^C NMR (100 MHz, DMSO) δ 20.1 (CH_3_), 43.8 (CH_2_), 116.7, 118.5, 120.3, 126.7, 127.2, 128.3, 130.6, 131.0, 131.4, 137.4, 137.5, 138.5, 152.8, 165.8 (CO), 167.5 (CO); C_17_H_14_ClN_3_O_2_; LC-ESI-MS (*m*/*z*): 328 [M+H]^+^; purity was confirmed by HPLC-UV (254 nm)-ESI-MS.

**General procedure for the synthesis of 2-substituted phenoxy*-N*-(4-oxo-1-(substituted phenyl)-1,4-dihydro quinazolin-6-yl)acetamide (7a–f).**

A mixture of substituted phenol (2.4 mmol), K_2_CO_3_ (2.4 mmol), and compound **6a** or **6b** (1.6 mmol) in CH_3_CN (50 mL) was refluxed for 8 h. The reaction was monitored by TLC. After the reaction was complete, the solvent was evaporated and a 4% NaOH solution (60 mL) was added to the residue to remove any remaining excess phenol as water-soluble sodium phenoxide salt. The product was separated and the precipitate was filtered, washed with water, and recrystallized to form methanol.

***N*-(4-Oxo-1-phenyl-1,4-dihydroquinazolin-6-yl)-2-(*p*-tolyloxy)acetamide (7a).**

Off-white powder, yield 80%; mp 220 °C; IR (KBr) νmax/cm^−1^: 3271 (NH), 3032 (CH-Ar), 2920 (CH-sp^3^), 1643 (CO); ^1^H NMR (400 MHz, DMSO-*d*_6_) δ 2.25 (s, 3H, CH_3_), 4.69 (S, 2H, CH_2_), 6.92 (s, 3H, Ar-H), 7.11 (s, 2H, Ar-H), 7.27–7.66 (m, 5H, Ar-H), 7.96 (s, 1H, Ar-H), 8.43 (s, 1H, Ar-H), 8.51 (s, 1H, Ar-H), 10.44 (s, 1H, NH, exchangeable by D_2_O); ^13^C NMR (100 MHz, DMSO) δ 20.6 (CH_3_), 67.8 (CH_2_), 112.0, 115.1 (2C), 117.2, 117.9, 126.6, 128.3, 130.3 (2C), 130.4, 130.8 (2C), 137.1, 138.1, 146.5, 147.9, 153.1, 156.2, 167.6 (CO); C_23_H_19_N_3_O_3_; LC-ESI-MS (*m*/*z*): 386 [M+H]^+^; purity was confirmed by HPLC-UV (254 nm)-ESI-MS.

**2-(4-Chlorophenoxy)-*N*-(4-oxo-1-phenyl-1,4-dihydroquinazolin-6-yl)acetamide (7b).**

Off-white powder, yield 80%, mp 262 °C; IR (KBr) νmax/cm^−1^: 3278 (NH), 3066 (CH-Ar), 2843 (CH-sp^3^), 1705 (CO), 1643 (CO); ^1^H NMR (400 MHz, DMSO-*d*_6_) δ 4.76 (s, 2H, CH_2_), 6.95 (s, 1H, Ar-H), 7.05 (s, 2H, Ar-H), 7.37 (s, 2H, Ar-H), 7.65 (s, 5H, Ar-H), 7.97 (s, 1H, Ar-H), 8.43 (s, 1H, Ar-H), 8.51 (s, 1H, Ar-H), 10.41 (s, 1H, NH, exchangeable by D_2_O); ^13^C NMR (100 MHz, DMSO) δ 67.7 (CH_2_), 117.0 (2C), 117.2, 117.9, 120.2, 125.5, 126.7, 128.2 (2C), 129.7 (2C), 130.5, 130.8 (2C), 137.0, 137.2, 137.9, 153.1, 157.0, 167.2 (CO), 168.7 (CO); C_22_H_16_ClN_3_O_3_; LC-ESI-MS (*m*/*z*): 406 [M+H]^+^; purity was confirmed by HPLC-UV (254 nm)-ESI-MS.

**2-(4-Chloro-3-methylphenoxy)-*N*-(4-oxo-1-phenyl-1,4-dihydroquinazolin-6-yl)acetamide (7c).**

White powder, yield 85%, mp 255 °C; IR (KBr) νmax/cm^−1^: 3275 (NH), 3066 (CH-Ar), 2978 (CH-sp^3^), 1685 (CO), 1643 (CO); ^1^H NMR (400 MHz, DMSO-*d*_6_) δ 2.31 (s, 3H, CH_3_), 4.73 (s, 2H, CH_2_), 6.89 (d, *J* = 12 Hz, 1H, Ar-H), 6.93 (d, *J* = 8 Hz, 1H, Ar-H), 7.05 (s, 1H, Ar-H), 7.33 (d, *J* = 8 Hz, 1H, Ar-H), 7.63–7.74 (m, 5H, Ar-H), 7.94 (d, *J* = 8 Hz, 1H, Ar-H), 8.45 (s, 1H, Ar-H), 8.50 (s, 1H, Ar-H), 10.42 (s, 1H, NH, exchangeable by D_2_O); ^13^C NMR (100 MHz, DMSO) δ 20.2 (CH_3_), 67.6 (CH_2_), 114.2, 117.2, 118.0, 120.2, 125.8, 126.7, 128.2 (2C), 130.0, 130.5, 130.8 (2C), 137.0, 137.2, 137.9, 153.1, 156.9, 167.3 (CO), 168.7 (CO); C_23_H_18_ClN_3_O_3_; LC-ESI-MS (*m*/*z*): 420 [M+H]^+^; purity was confirmed by HPLC-UV (254 nm)-ESI-MS.

***N*-(4-oxo-1-(*m*-tolyl)-1,4-dihydroquinazolin-6-yl)-2-(*p*-tolyloxy)acetamide (7d).**

Brown powder, 82%, mp 206 °C; IR (KBr) νmax/cm^−1^: 3282 (NH), 3062 (CH-Ar), 2958 (CH-sp^3^), 1705 (C=O); ^1^H NMR (400 MHz, DMSO-*d*_6_) δ 2.23 (s, 3H, CH_3_), 2.42 (s, 3H, CH_3_), 4.7 (s, 2H, CH_2_), 6.91 (d, *J* = 8 Hz, 2H, Ar-H), 6.95–6.99 (m, 1H, Ar-H), 7.11 (d, *J* = 8. Hz, 2H, Ar-H), 7.41–7.73 (m, 4H, Ar-H), 7.96 (d, *J* = 8 Hz, 1H, Ar-H), 8.43 (d, *J* = 4 Hz, Ar-H), 8.52 (s, 1H, Ar-H), 10.64 (s, 1H, NH, exchangeable by D_2_O); ^13^C NMR (100 MHz, DMSO) δ 20.1 (CH_3_), 20.5 (CH_3_), 67.8 (CH_2_), 115.0 (2C), 117.1, 118.0, 120.2, 126.6, 127.5, 128.6, 130.3 (2C), 130.5, 131.0 (2C), 135.2, 136.8, 137.1, 138.3, 153.0, 156.1, 167.6(CO), 168.5 (CO); C_24_H_21_N_3_O_3_; LC-ESI-MS (*m*/*z*): 400 [M+H]^+^; purity was confirmed by HPLC-UV (254 nm)-ESI-MS.

**2-(4-Chlorophenoxy)-*N*-(4-oxo-1-(*m*-tolyl)-1,4-dihydroquinazolin-6-yl)acetamide (7e).**

White powder, 79%, mp 255 °C; IR (KBr) νmax/cm^−1^: 3228 (NH), 3070 (CH-Ar), 2927 (CH-sp^3^), 1701 (C=O), 1643 (CO); ^1^H NMR (400 MHz, DMSO-*d*_6_) δ 2.42 (s, 3H, CH_3_), 4.80 (s, 2H, CH_2_), 6.94 (d, *J* = 16 Hz, 1H, Ar-H), 7.04 (d, *J* = 8 Hz, 2H, Ar-H), 7.35 (d, *J* = 8 Hz, 2H, Ar-H), 7.41–7.74 (m, 4H, Ar-H), 7.97 (s, 1H, Ar-H), 8.42 (s, 1H, Ar-H), 8.52 (s, 1H, Ar-H), 10.86 (s, 1H, NH, exchangeable by D_2_O); ^13^C NMR (100 MHz, DMSO) δ 21.3 (CH_3_), 67.7 (CH_2_), 117.0, 117.1, 118.0, 120.2, 125.2, 125.4, 126.6, 127.5, 128.6, 129.7, 130.5, 131.0, 135.2, 136.8, 137.0, 137.1, 138.3, 152.9, 157.1, 167.1 (CO), 168.5 (CO); C_23_H_18_ClN_3_O_3_; LC-ESI-MS (*m*/*z*): 420 [M+H]^+^; purity was confirmed by HPLC-UV (254 nm)-ESI-MS.

**2-(4-Chloro-3-methylphenoxy)-*N*-(4-oxo-1-(*m*-tolyl)-1,4-dihydroquinazolin-6-yl)acetamide (7f).**

Pale yellow powder, yield 85%, mp = 269 °C; IR (KBr) νmax/cm^−1^: 3278(NH), 3066 (CH-Ar), 2970 (CH-sp^3^), 1705 (CO), 1643 (CO); ^1^H NMR (400 MHz, DMSO-*d*_6_) δ 2.32 (s, 3H, CH_3_), 2.42 (s, 3H, CH_3_), 4.73 (s, 2H, CH_2_), 6.89 (d, *J* = 4 Hz, 1H, Ar-H), 6.97 (m, 1H, Ar-H), 7.05 (s, 1H, Ar-H), 7.33 (d, *J* = 8 Hz, 1H, Ar-H), 7.42–7.73 (m, 4H, Ar-H), 7.95 (d, *J* = 12 Hz, 1H, Ar-H), 8.44 (d, *J* = 4 Hz, 1H, Ar-H), 8.49 (s, 1H, Ar-H), 10.43 (s, 1H, NH, exchangeable by D_2_O); ^13^C NMR (100 MHz, DMSO) δ 20.2 (CH_3_), 21.2 (CH_3_), 67.6 (CH_2_), 114.2, 117.2, 118.0, 118.1, 120.1, 125.1, 125.8, 126.8, 127.4, 128.4, 130.0, 130.6, 131.2, 136.9, 137.1, 137.8, 140.7, 153.0, 156.8, 167.4 (CO), 168.8 (CO); C_24_H_20_ClN_3_O_3_; LC-ESI-MS (*m*/*z*): 434 [M+H]^+^; purity was confirmed by HPLC-UV (254 nm)-ESI-MS.

**General procedure for the synthesis of 2-methyl-6-nitro-1-(substituted phenyl)quinazolin-4(1*H*)-one (8a, 8b).**

A mixture of compound **3a** or **3b** (5 mmol), acetic anhydride (10 mL) and sulfuric acid (1mL) was refluxed for 8 h (monitored by TLC). After the reaction was complete, it was poured into ice water. The product was separated out. The precipitated solid was filtrated off, washed with hot hexane, and dried in vacuo.

**2-Methyl-6-nitro-1-phenylquinazolin-4(1*H*)-one (8a).**

Greenish yellow powder, yield 80%, mp 260–262 °C; IR (KBr) νmax/cm^−1^: 3062 (CH-Ar), 1651 (CO), 1543, 1330 (NO_2_); ^1^H NMR (400 MHz, DMSO-*d_6_*): δppm = 2.18 (s, 3H, CH_3_), 6.77 (d, *J* = 12 Hz, 1H, Ar-H), 7.66–7.82 (m, 5H, Ar-H), 8.40 (s, 1H, Ar-H), 8.80 (s, 1H, Ar-H); ^13^C NMR (100 MHz, DMSO) δ 24.9 (CH_3_), 118.9, 119.2, 122.6, 123.3, 128.5, 128.9, 129.9, 131.0, 131.4, 137.9, 144.6, 146.6, 163.5, 167.55 (CO); C_15_H_11_N_3_O_3_; LC-ESI-MS (*m*/*z*): 282 [M+H]^+^; purity was confirmed by HPLC-UV (254 nm)-ESI-MS.

**2-Methyl-6-nitro-1-(*m*-tolyl)quinazolin-4(1*H*)-one (8b).**

Greenish yellow powder, 83%, mp 258–260 °C IR (KBr) νmax/cm^−1^: 3059 (CH-Ar), 2993 (CH-sp^3^), 1658 (C=O), 1543, 1334 (NO_2_); ^1^H NMR (400 MHz, DMSO-*d*_6_) δ 2.18 (s, 3H, CH_3_), 2.44 (s, 3H, CH_3_), 6.82 (dd, *J* = 17.2 Hz and 10.8 Hz, 1H, Ar-H), 7.45–7.82 (m, 4H, Ar-H), 8.35–8.40 (m, 1H, Ar-H), 8.71 (s, 1H, Ar-H); ^13^C NMR (100 MHz, DMSO) δ 21.3(CH_3_), 24.9(CH_3_), 118.7, 119.3, 123.2, 125.8, 128.1, 128.4, 129.1, 131.1, 131.6, 137.8, 141.3, 144.5, 146.5, 163.6 (CO); C_16_H_13_N_3_O_3_, LC-ESI-MS (*m*/*z*): 296 [M+H]^+^; purity was confirmed by HPLC-UV (254 nm)-ESI-MS.

**General procedure for the synthesis of 6-amino-2-methyl-1-(substituted phenyl)quinazolin-4(1*H*)-one (9a, 9b).**

A mixture of compound **8a** or **8b** (5 mmol), stannous chloride dihydrate (5.6 g, 25 mmol), and conc. HCl (5 mL) in 15 mL ethyl acetate was stirred at room temperature for 2 h. The reaction was monitored with TLC. After the reaction was complete, the reaction mixture was diluted with a mixture of ethyl acetate and methanol (3:1) (200 mL) and pH was adjusted to 7 through the addition of sodium carbonate powder. The solution mixture was filtered, and the organic filtrate was dried over sodium sulphate. The filtration of sodium sulphate and the evaporation of the solvent under a vacuum afforded the crude products. The obtained residue was purified by silica gel chromatography using a hexane/ethyl acetate mixture (2:1) to produce the desired compounds.

**6-Amino-2-methyl-1-phenylquinazolin-4(1*H*)-one (9a).**

White powder, 69%, mp 268–270 °C; IR (KBr) νmax/cm^−1^: 3417, 3332 (NH_2_), 1708 (C=O); ^1^H NMR (400 MHz, DMSO-*d*_6_) δ 2.07 (s, 3H, CH_3_), 5.51(brs, 2H, NH_2_, exchangeable by D_2_O), 6.28 (d, *J* = 8 Hz, 1H, Ar-H), 6.89 (s, 1H, Ar-H), 7.22 (s, 1H, Ar-H), 7.52 –7.73 (m, 5H, Ar-H); ^13^C NMR (100 MHz, DMSO) δ 24.0 (CH_3_), 107.8, 118.1, 120.4, 121.9, 128.8, 130.4, 131.1, 134.0, 138.3, 147.5, 158.8, 159.5, 168.8, 175.4 (CO); C_15_H_13_N_3_O; LC-ESI-MS (*m*/*z*): 252 [M+H]^+^; purity was confirmed by HPLC-UV (254 nm)-ESI-MS.

**6-Amino-2-methyl-1-(*m*-tolyl)quinazolin-4(1*H*)-one (9b).**

Yellow powder, 67%, mp 270–273 °C IR (KBr) νmax/cm^−1^: 3402, 3321 (NH_2_), 3066 (CH-Ar), 2978 (CH-sp^3^), 1635 (C=O); ^1^H NMR (400 MHz, DMSO-*d*_6_) δ 2.08 (s, 3H, CH_3_), 2.40 (s, 3H, CH_3_), 5.52 (brs, 2H, NH_2,_ exchangeable by D_2_O), 6.35 (d, *J* = 8 Hz, 1H, Ar-H), 6.91 (d, *J* = 8 Hz, 1H, Ar-H), 7.21 (s, 1H, Ar-H), 7.38 (d, *J* = 8 Hz, 1H, Ar-H), 7.54 (s, 1H, Ar-H), 7.00 (d, *J* = 12 Hz, 1H, Ar-H); ^13^C NMR (100 MHz, DMSO) δ 20.1 (CH_3_), 24.1 (CH_3_), 107.9, 118.1, 120.5, 121.8, 128.1, 131.3, 133.8, 135.2, 137.2, 138.7, 147.5, 158.6, 168.6, 175.9 (C=O); C_16_H_15_N_3_O; LC-ESI-MS (*m*/*z*): 266 [M+H]^+^; purity was confirmed by HPLC-UV (254 nm)-ESI-MS.

**General procedure for the synthesis of 2-chloro-*N*-(2-methyl-4-oxo-1-(substituted phenyl)-1,4-dihydroquinazolin-6-yl)acetamide derivatives (10a, 10b).**

Chloroacetyl chloride (0.5 mL, 6.3 mmol) was slowly added to a magnetically stirred solution of compound **9a** or **9b** (4.2 mmol) and K_2_CO_3_ (0.873 g, 6.3 mmol) in CH_2_Cl_2_ (10 mL). The reaction mixture was stirred at room temperature and monitored by TLC. After the reaction was complete, the solvents were evaporated, and ice water (100 mL) was added to the residue. The product was separated out. The precipitate was filtered, washed with water, and left to dry in vacuo.

**2-Chloro-*N*-(2-methyl-4-oxo-1-phenyl-1,4-dihydroquinazolin-6-yl)acetamide (10a).**

Yellow powder, 83%, mp 285 °C; IR (KBr) νmax/cm^−1^: 3271(NH), 3074 (CH-Ar), 2962 (CH-sp^3^), 1701 (C=O); ^1^H NMR (400 MHz, DMSO-*d*_6_) δ 2.14 (s, 3H, CH_3_), 4.28 (s, 2H, CH_2_), 6.6 (d, *J* = 16 Hz, 1H, Ar-H), 7.56–7.76 (m, 6H, Ar-H), 8.44 (s, 1H, Ar-H), 10.67 (s, 1H, NH, exchangeable by D_2_O); ^13^C NMR (100 MHz, DMSO) δ 23.7 (CH_3_), 43.8 (CH_2_), 116.4, 118.6, 119.6, 126.5, 128.6, 130.8, 130.9 (2C), 131.3, 136.9, 137.5, 138.7, 161.4, 165.7 (CO), 166.8 (CO); C_17_H_14_ClN_3_O_2_; LC-ESI-MS (*m*/*z*): 328 [M+H]^+^; purity was confirmed by HPLC-UV (254 nm)-ESI-MS.

**2-Chloro-*N*-(2-methyl-4-oxo-1-(*m*-tolyl)-1,4-dihydroquinazolin-6-yl)acetamide (10b).**

Reddish white powder, 88%, mp 280 °C; IR (KBr) νmax/cm^−1^: 3271 (NH), 3073 (CH-Ar), 2958 (CH-sp^3^), 1701 (C=O); ^1^H NMR (400 MHz, DMSO-*d*_6_) δ 2.14 (s, 3H, CH_3_), 2.39 (s, 3H, CH_3_), 4.30 (s, 2H, CH_2_), 6.63 (d, *J* = 16 Hz, 1H, Ar-H), 7.33–7.78 (m, 5H, Ar-H), 8.42 (s, 1H, Ar-H), 10.85 (s, 1H, NH, exchangeable by D_2_O); ^13^C NMR (100 MHz, DMSO) δ 20.1 (CH_3_), 24.3 (CH_3_), 43.8 (CH_2_), 116.5, 118.2, 119.3, 126.3, 126.7, 128.0, 131.3, 131.4, 135.5, 136.5, 136.6, 139.0, 161.1, 165.6 (CO), 168.4 (CO); C_18_H_16_ClN_3_O_2_; LC-ESI-MS (*m*/*z*): 342 [M+H]^+^; purity was confirmed by HPLC-UV (254 nm)-ESI-MS.

**General procedure for the synthesis of 2-substituted phenoxy-*N*-[2-methyl-4-oxo-1-(substituted phenyl)-1,4-dihydro quinazolin-6-yl]acetamide (11a–f).**

A mixture of substituted phenol (2.4 mmol), K_2_CO_3_ (0.33 g, 2.4 mmol), and compound **10a** or **10b** (1.6 mmol) in CH_3_CN (50 mL) was refluxed for 8 h. The reaction was monitored by TLC. After the reaction was complete, the solvent was evaporated, and a 4% NaOH solution (60 mL) was added to the residue to remove any remaining excess phenol as water-soluble sodium phenoxide salt. The product was separated out. The precipitate was filtered, washed with water, and recrystallized to form methanol.

***N*-(2-Methyl-4-oxo-1-phenyl-1,4-dihydroquinazolin-6-yl)-2-(*p*-tolyloxy)acetamide (11a).**

White powder, yield 85%, mp 268 °C; IR (KBr) νmax/cm^−1^: 3271(NH), 3066 (CH-Ar), 2970 (CH-sp^3^), 1705 (CO); ^1^H NMR (400 MHz, DMSO-*d*_6_) δ 2.13 (s, 3H, CH_3_), 2.23 (s, 3H, CH_3_), 4.69 (s, 2H, CH_2_), 6.55 (d, *J* = 10 Hz, 1H, Ar-H), 6.90 (d, *J* = 8 Hz, 2H, Ar-H), 7.11 (d, *J* = 6.4 Hz, 2H, Ar-H), 7.57–7.78 (m, 5H, Ar-H), 7.85 (d, *J* = 12 Hz, 1H, Ar-H), 8.46 (s, 1H, Ar-H), 10.47 (s, 1H, NH, exchangeable by D_2_O); ^13^C NMR (100 MHz, DMSO) δ 20.5 (CH_3_), 24.4 (CH_3_), 67.6 (CH_2_), 115.0 (3C), 116.9, 117.9, 119.4, 126.5, 128.9 (2C), 130.3 (2C), 130.6, 131.2 (2C), 136.3, 138.1, 139.1 156.0, 161.1, 167.6 (CO), 168.5 (CO); C_24_H_21_N_3_O_3_; LC-ESI-MS (*m*/*z*): 400 [M+H]^+^; purity was confirmed by HPLC-UV (254 nm)-ESI-MS.

**2-(4-Chlorophenoxy)-*N*-(2-methyl-4-oxo-1-phenyl-1,4-dihydroquinazolin-6-yl)acetamide (11b).**

White powder, yield 87%, mp 254 °C; IR (KBr) νmax/cm^−1^: 3267 (NH), 3070 (CH-Ar), 2974 (CH-sp^3^), 1705 (CO); ^1^H NMR (400 MHz, DMSO-*d*_6_) δ 2.12 (s, 3H, CH_3_), 4.76 (s, 2H, CH_2_), 6.56 (d, *J* = 10 Hz, 1H, Ar-H), 7.05 (d, *J* = 12 Hz, 2H, Ar-H), 7.36 (d, *J* = 8 Hz, 2H, Ar-H), 7.57–7.78 (m, 5H, Ar-H), 7.84 (d, *J* = 12 Hz, 1H, Ar-H), 8.47 (s, 1H, Ar-H), 10.52 (s, 1H, NH, exchangeable by D_2_O); ^13^C NMR (100 MHz, DMSO) δ 24.4 (CH_3_), 67.6 (CH_2_), 117.0 (2C), 117.9, 119.4, 125.5, 126.5, 128.9 (2C), 129.7 (2C), 130.6, 131.2 (2C), 136.3, 138.1, 139.0, 157.0, 161.2, 167.2, 168.4 (CO), 169.6 (CO); C_23_H_18_ClN_3_O_3_; LC-ESI-MS (*m*/*z*): 420 [M+H]^+^; purity was confirmed by HPLC-UV (254 nm)-ESI-MS.

**2-(4-Chloro-3-methylphenoxy)-*N*-(2-methyl-4-oxo-1-phenyl-1,4-dihydroquinazolin-6-yl)acetamide (11c).**

White powder, yield 80%, mp 271 °C; IR (KBr) νmax/cm^−1^: 3267 (NH), 3074 (CH-Ar), 2927 (CH-sp^3^), 1701 (CO); ^1^H NMR (400 MHz, DMSO-*d*_6_) δ 2.13 (s, 3H, CH_3_), 2.30 (s, 3H, CH_3_), 4.71 (s, 2H, CH_2_), 6.53 (s, 1H, Ar-H), 6.89 (s, 1H, Ar-H), 7.04 (s, 1H, Ar-H), 7.34 (s, 1H, Ar-H), 7.60–7.69 (m, 5H, Ar-H), 7.82 (s, 1H, Ar-H), 8.48 (s, 1H, Ar-H), 10.32 (s, 1H, NH, exchangeable by D_2_O); ^13^C NMR (100 MHz, DMSO) δ 20.3 (CH_3_), 24.5 (CH_3_), 67.7 (CH_2_), 114.3, 116.9, 117.9, 118.0, 119.4, 125.8, 126.4, 128.9 (2C), 130.0, 130.6, 131.2 (2C), 136.3, 137.0, 138.2, 139.0, 157.0, 160.9, 167.2(CO), 168.3(CO); C_24_H_20_ClN_3_O_3_; LC-ESI-MS (*m*/*z*): 434 [M+H]^+^; purity was confirmed by HPLC-UV (254 nm)-ESI-MS.

***N*-(2-Methyl-4-oxo-1-(*m*-tolyl)-1,4-dihydroquinazolin-6-yl)-2-(*p*-tolyloxy)acetamide (11d).**

White powder, yield 85%, mp 270 °C; IR (KBr) νmax/cm^−1^: 3221 (NH), 3074 (CH-Ar), 2962 (CH-sp^3^), 1701 (CO); ^1^H NMR (400 MHz, DMSO-*d*_6_) δ 2.14 (s, 3H, CH_3_), 2.23 (s, 3H, CH_3_), 2.41 (s, 3H, CH_3_), 4.66 (s, 2H, CH_2_), 6.62 (s, 1H, Ar-H), 6.91 (s, 2H, Ar-H), 7.11 (s, 2H, Ar-H), 7.47–7.77 (m, 4H, Ar-H), 7.85 (s, 1H, Ar-H), 8.46 (s, 1H, Ar-H), 10.38 (s, 1H, NH, exchangeable by D_2_O); ^13^C NMR (100 MHz, DMSO) δ 20.2 (CH_3_), 20.5 (CH_3_), 24.5 (CH_3_), 67.8 (CH_2_), 115.0 (2C), 116.9, 117.9, 119.4, 126.4, 128.2, 130.3 (2C), 130.4, 131.4, 131.6, 135.4, 136.5, 137.0, 138.8, 156.2, 160.7, 167.5 (CO), 168.2 (CO); C_25_H_23_N_3_O_3_; LC-ESI-MS (*m*/*z*): 414 [M+H]^+^; purity was confirmed by HPLC-UV (254 nm)-ESI-MS.

**2-(4-Chlorophenoxy)-*N*-(2-methyl-4-oxo-1-(*m*-tolyl)-1,4-dihydroquinazolin-6-yl)acetamide (11e).**

White powder, yield 85%, mp 283 °C; IR (KBr) νmax/cm^−1^: 3221 (NH), 3074 (CH-Ar), 2927 (CH-sp^3^), 1701 (CO); ^1^H NMR (400 MHz, DMSO-*d*_6_) δ 2.14 (s, 3H, CH_3_), 2.42 (s, 3H, CH_3_), 4.73 (s, 2H, CH_2_), 6.64 (d, *J* = 12 Hz, 1H, Ar-H), 7.04 (d, *J* = 8 Hz, 2H, Ar-H), 7.36 (d, *J* = 8 Hz, 2H, Ar-H), 7.46–7.75 (m, 4H, Ar-H), 7.82 (d, *J* = 8.0 Hz, 1H, Ar-H), 8.45 (s, 1H, Ar-H), 10.40 (s, 1H, NH, exchangeable by D_2_O); ^13^C NMR (100 MHz, DMSO) δ 20.2, 24.5, 67.8, 116.9(2C), 117.0, 118.0, 119.4, 121.8, 125.4, 126.3, 128.3, 129.7(2C), 131.4, 131.7, 135.4, 137.00, 138.8, 138.9, 157.1, 160.7, 167.0 (CO), 168.2(CO); C_24_H_20_ClN_3_O_3_; LC-ESI-MS (*m*/*z*): 434 [M+H]^+^; purity was confirmed by HPLC-UV (254 nm)-ESI-MS.

**2-(4-Chloro-3-methylphenoxy)-*N*-(2-methyl-4-oxo-1-(*m*-tolyl)-1,4-dihydroquinazolin-6-yl)acetamide (11f).**

White powder, yield 87%, mp 290 °C; IR (KBr) νmax/cm^−1^: 3221 (NH), 3074 (CH-Ar), 2927 (CH-sp^3^), 1701 (CO), 1643 (CO); ^1^H NMR (400 MHz, DMSO-*d*_6_) δ 2.14 (s, 3H, CH_3_), 2.30 (s, 3H, CH_3_), 2.42 (s, 3H, CH_3_), 4.71 (s, 2H, CH_2_), 6.63 (d, *J* = 8 Hz, 1H, Ar-H), 6.87 (d, *J* = 8 Hz, 1H, Ar-H), 7.03 (s, 1H, Ar-H), 7.32 (d, *J* = 8 Hz, 1H, Ar-H), 7.39–7.75 (m, 4H, Ar-H), 7.83 (d, *J* = 8 Hz, 1H, Ar-H), 8.45 (s, 1H, Ar-H), 10.37 (s, 1H, NH, exchangeable by D_2_O); ^13^C NMR (100 MHz, DMSO) δ 20.2 (CH_3_), 21.3 (CH_3_), 24.4 (CH_3_), 67.7 (CH_2_), 114.3, 116.4, 116.9, 118.0, 119.4, 125.8, 126.0, 128.2, 130.0, 131.6, 135.4, 136.4, 136.9, 137.0, 138.9, 138.9, 157.0, 160.9, 165.4, 167.2 (CO), 168.2 (CO); C_25_H_22_ClN_3_O_3_; LC-ESI-MS (*m*/*z*): 448 [M+H]^+^; purity was confirmed by HPLC-UV (254 nm)-ESI-MS.

### 2.2. Cell Culture

The Huh5-2 stable cell line, containing the HCV subgenomic reporter replicon of GT 1b I389luc-ubi-neo/NS3-3′/Con1/5.1 (strain Con1), has been previously described [52] and was kindly provided by Prof. R. Bartenschlager (Heidelberg University, Germany). Huh7.5-3a and Huh7.5-4a cell lines have been previously reported [53] and harbor the subgenomic reporter replicons of HCV GT 3a (S52) S52-SG(Feo)(AII) and GT 4a (ED43) ED43-SG(Feo)(VYG), respectively, that were kindly provided by Prof. C.M. Rice (The Rockefeller University, New York, NY, USA) [54]. The subgenomic reporter replicon DENV2 16,681 pD2-hRUPac (kindly provided by Prof. C.M. Rice) has been previously described [55] and was used to construct the stable cell line Huh7-D2 [53]. Cells were cultured in Dulbecco’s modified minimal essential medium containing high glucose (25 mM) (Invitrogen), supplemented with 2 mM L-glutamine, 0.1 mM non-essential amino acids, 100 U/mL penicillin, 100 µg/mL streptomycin, and 10% (*v*/*v*) fetal calf serum (referred to as complete DMEM). Complete DMEM was supplemented with G418 at 500 μg/mL for Huh5-2, 750 μg/mL for Huh7.5-3a, and 350 μg/mL for Huh7.5-4a, or puromycin at 0.5 μg/mL for Huh7-D2.

### 2.3. Cytotoxicity Assay

Cytotoxicity of the compounds was determined by the alamarBlue^®^ reduction bioassay, which is based on alamarBlue^®^ reduction by cellular enzymes involved in the oxidation-reduction reaction. Specifically, 10^4^ replicon cells were seeded in 96-well flat-bottom plates in a total volume of 100 μL of complete DMEM per well. After 24 h, cells were incubated with the compounds for 3 days at 37 °C (5% CO_2_). Cells treated with the solvent DMSO were used as the control. Ten microliters of alamarBlue^®^ were added to each well, and cells were further incubated at 37 °C for 3 h. Absorbance was read at 570 nM (reduced) and 600 nm (oxidized) using a plate reader. The percent difference in dye reduction between treated and control cells was calculated and used for the determination of the compound concentration causing 50% cell death (CC_50_). CC_50_ values were determined by nonlinear regression analysis after converting the drug concentrations into log-X using the Prism 5.0 software (GraphPad Software Inc., San Diego, CA, USA).

### 2.4. Antiviral Assay

An antiviral assay was performed by seeding 10^4^ replicon cells per well in a 96-well flat bottom plate. The medium was exchanged with serial dilutions of the test compounds in complete DMEM. Compounds were diluted in DMSO, and the final concentration of DMSO in complete DMEM was 0.2%. Cells were cultured at 37 °C (5% CO_2_) and lysed after 3 days. Viral replication levels were determined by measuring Firefly luciferase (F-Luc) activity in cell lysates by using the Luciferase Assay System (Promega), as recommended by the manufacturer. Measurements were performed with a GloMax 20/20 single-tube luminometer (Promega) for 10 s. F-Luc activity was normalized to the total intracellular protein amount as determined by the Bradford assay (Pierce). Relative luminescence units (RLUs) were calculated as the percentage of the respective values from DMSO-treated control cells. The median effective concentration (EC_50_) was defined as the concentration of the compound that reduced the luciferase signal by 50%. EC_50_ values were determined by nonlinear regression analysis after converting the drug concentrations into log-X using the Prism 5.0 software (GraphPad Software Inc.). Daclatasvir used in the drug combination experiments was kindly provided by Dr. Marc Windisch (Institute Pasteur Korea). The coefficient of drug interaction in these experiments was calculated as follows: CDI = AB/(A × B) where AB is the ratio of the viral replication (luciferase activity levels) resulting from cells treated by the 2-drug combination, normalized to the control group (cells treated with the solvent DMSO), which is set to 1, and A or B is the ratio of the viral replication resulting from the single drug, normalized to the control group.

### 2.5. Total RNA Extraction and Quantification of Viral Replicons

Total RNA was extracted from replicon cells seeded in 12-well plates using the NucleoZOL reagent (Macherey-Nagel) based on the manufacturer’s recommendation and subjected to reverse-transcription (RT) for cDNA synthesis. cDNA was then used as a template for the quantitative real-time polymerase chain reaction (qPCR). RT was performed using Moloney Murine Leukemia Virus (MMLV) reverse transcriptase (Promega) and reverse primers specific for Con1 IRES (5’-GGATTCGTGCTCATGGTGCA-3’) and the housekeeping gene YWHAZ (5’-GGATGTGTTGGTTGCATTTCCT-3’). For the qPCR reactions, Con1 IRES-specific primers (forward: 5’-GGCCTTGTGGTACTGCCTGATA-3’ and reverse: 5’-GGATTCGTGCTCATGGTGCA-3’) and the Luna Universal qPCR Master Mix (NEB) were used. YWHAZ mRNA was used for normalization (forward: 5’-GCTGGTGATGACAAGAAAGG-3’ and reverse: 5’-GGATGTGTTGGTTGCATTTCCT-3’). 

### 2.6. Gel Electrophoresis and Western Blot Analysis

Denaturing SDS-polyacrylamide gel electrophoresis and Western blotting were performed as previously described [56]. The following antibodies were used: HCV NS5A (9E10) monoclonal antibody at a dilution of 1:2000 (kindly provided by Prof. C.M. Rice), the β-actin monoclonal antibody at 1:6000 (Merck-Millipore), and the secondary anti-mouse horseradish peroxidase-conjugated antibody at 1:2000 (Cell Signaling).

### 2.7. Statistical Analysis

In the diagrams, bars represent the mean values of three independent experiments in triplicate. Error bars represent standard deviation. Only results subjected to statistical analysis using Student’s *t*-test with *p* ≤ 0.05 were considered statistically significant and presented. Statistical calculations were performed with Excel Microsoft Office^®^.

### 2.8. Determination of Water Solubility of Compounds ***11a*** and ***11b***

In a capped 5 mL polypropylene tube, an excess quantity of compounds **11a** or **11b** was added to 1mL aliquot of deionized water. A stirrer magnet was placed, the tube was capped and rapped with aluminum foil then placed on a stirring plate at an ambient temperature. The suspension was agitated at an ambient temperature for 24 h after which it was centrifuged at 5000 rpm for 5 min. The clear supernatant was pipetted and filtered through a 0.2 µm syringe filter disc and then transferred to an HPLC vial. A 20 µL aliquot was injected into an HPLC system equipped with a UV detector set at 254nm. Elution was achieved isochratically on an RP C18 column (250 × 4.5 mm) with 90% methanol in water as a mobile phase. The column temperature was thermostatically controlled at 40 °C and the mobile phase flow rate was 1 mL/min. Linear regression analysis of integrated peak areas versus the concentration of a series of standard solutions of each compound in methanol (1–500 µg/mL) was used to prepare the calibration curve from which the concentrations of the solubility assessment samples were estimated. 

### 2.9. Computational Chemistry

All protein and ligand preparation, docking, and free energy calculations, as well as the analysis of the poses, were conducted using the Schrödinger platform, LLC, New York, NY, USA, release 2019-1. Ligand preparation was performed with the default protocol of LigPrep using Epik [57,58] to generate protonation states at pH = 7.4 ± 2.0. Protein preparation was performed using the default protocol of Protein Preparation Wizard, using Prime to fill in missing side chains and loops, capping termini, and using Epik to generate protonation states at pH = 7.4 ± 2.0. The docking grid was created using the Receptor Grid Generation module of Glide at default settings. Rigid docking was performed with the default protocol of Glide, with the use of XP scoring (extra precision), performing post-docking minimization and applying strain-correction terms. Induced-fit docking was performed with the default protocol of the Induced Fit module of Glide, with the use of XP scoring (extra precision). The MMGBSA free energy of binding calculation was performed using the default protocol of Prime. The structure–activity relationships (SARs), such as hydrogen bonds, π–π interactions, cation–π interactions, and halogen bonds were identified automatically using the default settings of Maestro. Only the atom/ring distances were measured manually.

## 3. Results and Discussion

### 3.1. Chemistry

**Scheme 1:**
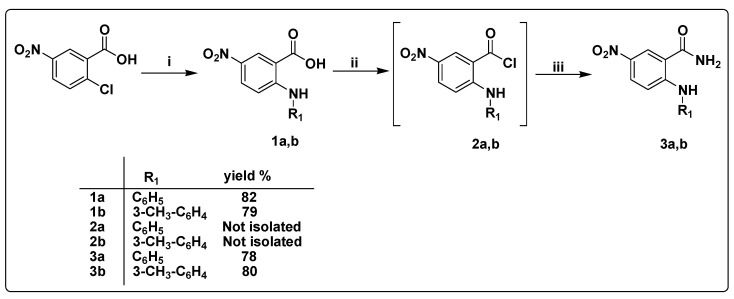
Synthesis of the 5-nitro-2-(substitutedphenylamino)benzamide derivatives **3a**,**b**. (i) Aromatic amine, fusion, 120 °C, 8–10 h; (ii) PCl_5_, CH_2_Cl_2_, reflux, 6 h; (iii) 25% aq. NH_4_OH, r.t., 1 h.

**Scheme 2:**
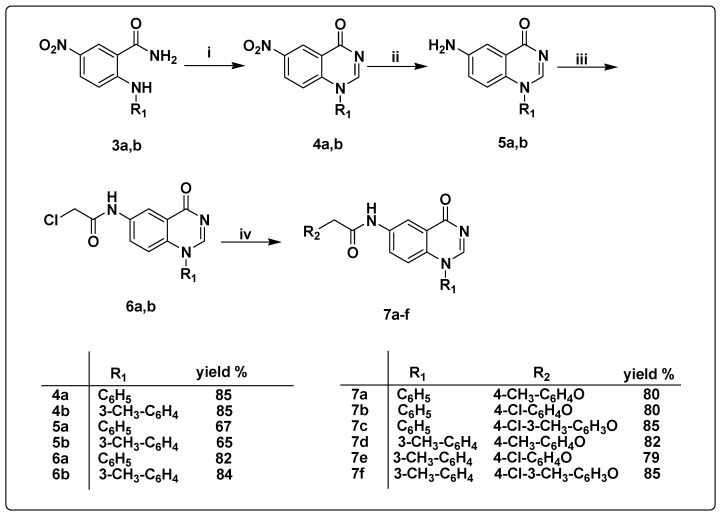
Synthesis of the target quinazoline derivatives **7a**–**f** (i) triethyl orthoformate, sulfuric acid, fusion, 100 °C, 8h; (ii) SnCl_2_, HCl, r.t, 2h; (iii) chloroacetyl chloride, CH_2_Cl_2_, K_2_CO_3_, r.t, 3h; (iv) substituted phenols, K_2_CO_3_, CH_3_CN, 90 °C, 8h.

**Scheme 3:**
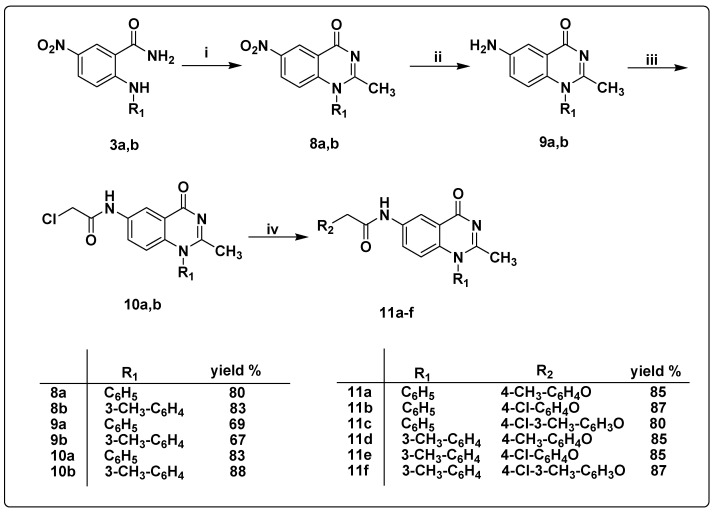
Synthesis of the target quinazoline derivatives **11a**–**f** (i) acetic anhydride, sulfuric acid, fusion, 100 °C, 8h; (ii) SnCl_2_, HCl, r.t, 2h; (iii) chloroacetyl chloride, CH_2_Cl_2_, K_2_CO_3_, r.t, 3h; (iv) substituted phenols, K_2_CO_3_, CH_3_CN, 90 °C, 8h.

The synthetic strategies designed for the preparation of 5-nitro-2-(substituted phenylamino)benzamide derivatives **3a,b**, 2-substituted phenoxy*-N*-(4-oxo-1-(substituted phenyl)-1,4-dihydro quinazolin-6-yl)acetamide derivatives **7a**–**f**, and 2-substituted phenoxy*-N*-(2-methyl-4-oxo-1-(substitutedphenyl)-1,4-dihydroquinazolin-6-yl)acetamide **11a**–**f** are illustrated in Scheme 1, Scheme 2 and Scheme 3. The starting material 5-nitro-2-chlorobenzoic acid was fused with aromatic amines, namely, aniline and 3-methylaniline, at 120 °C to afford 5-nitro-2-(phenylamino)-benzoic acid **1a** [48] and 5-nitro-2-(*m*-tolylamino)benzoic acid **1b** [49], respectively. The formation of compounds **1a,b** is believed to proceed via an S_N_Ar addition–elimination reaction [48]. 

5-Nitro-2-(substituted phenylamino)benzoyl chlorides **2a,b** were prepared via the reaction of 5-nitro-2-(substitutedphenylamino)benzoic acids **1a,b,** respectively, with PCl_5_ in methylene chloride under reflux [59,60]. Instability of the prepared compounds **2a,b** and partial hydrolysis to their corresponding carboxylic acids were observed and confirmed on TLC. Therefore, immediately after the formation of compounds **2a,b** and without separation and purification, these were transformed into compounds **3a,b**. 

Thus, the solution of compounds **2a,b** in methylene chloride was added dropwise to a 25% aqueous ammonium hydroxide solution to afford compounds **3a,b** in excellent yields [50,51,61]. The IR spectrum of compound **3b** showed three absorption peaks at 3437, 3344, and 3193 cm^−1^ corresponding to NH_2_ and NH moieties. Furthermore, the ^1^H NMR spectra of compound **3b** showed D_2_O exchangeable singlet signals at 10.89 ppm corresponding to aniline NH proton along with two D_2_O exchangeable singlet signals at 7.75 and 8.51 ppm due to magnetically inequivalent protons of amide moiety. Furthermore, ^13^C NMR spectra showed a typical signal for a carbonyl carbon at 170.4 ppm corresponding to the amide moiety.

Rad-Moghadam and Khajavi recently reported the synthesis of 2-subtituted-4(3*H*)-quinazolinones under microwave conditions. A mixture of anthranilic acid, ammonium acetate, and triethyl orthoformate was subjected to microwave irradiation under solvent-free conditions to produce 2-substituted-4(3*H*)-quinazolinones [62]. Later, Wang et al. reported a similar approach for the synthesis of a series of 1-substituted 4(1*H*)-quinazolinones [63]. Compounds **3a,b** were fused with triethyl orthoformate and a catalytic amount of sulfuric acid to afford 6-nitro-1-phenylquinazolin-4(1*H*)-one **4a** and 6-nitro-1-(*m*-tolyl)quinazolin-4(1*H*)-one **4b**, respectively, in high yields [64]. The IR spectrum confirmed the formation of compounds **4a,b** via the disappearance of OH and NH absorption bands at 3437, 3344, and 3193 cm^−1^ and the appearance of a peak at 1670 cm^−1^ owing to the carbonyl group at C-4 of the quinazoline ring. Moreover, the ^1^H NMR spectra confirmed the formation of compounds **4a,b** through the disappearance of three D_2_O-exchangeable singlet signals at 7.78, 8.54, and 10.99 ppm along with the appearance of a singlet signal at 8.81 ppm corresponding to one proton at C-2 carbon of the quinazoline ring. 

In a similar manner, 2-methyl-6-nitro-1-phenylquinazolin-4(1*H*)-one **8a** and 2-methyl-6-nitro-1-(*m*-tolyl)quinazolin-4(1*H*)-one **8b** were obtained from fusing acetic anhydride with compounds **3a** and **3b**, respectively, in the presence of a catalytic amount of sulfuric acid [65]. The ^1^H NMR spectra of compound **8a** showed the disappearance of D_2_O-exchangeable signals at 7.78, 8.54, and 10.99 ppm and the presence of a singlet signal at 2.18 ppm corresponding to CH_3_ protons at C-2 carbon of quinazoline ring. 

Compounds **4a,b** and **8a,b** were subjected to reduction via a reaction with stannous chloride dihydrate, SnCl_2_, and 2H_2_O in the presence of a catalytic amount of HCl at room temperature for 2 h, yielding compounds **5a,b** and **9a,b,** respectively [66]. The IR spectra of compounds **5a,b** and **9a,b** showed a characteristic forked peak at 3425 and 3336 cm^−1^ for compounds **5a-b**, 3417 and 3332 cm^−1^ for compound **9a,** and 3402 and 3321 cm^−1^ for compound **9b,** attributed to their amino groups. The ^1^H NMR spectra of compounds **5a,b** and **9a,b** revealed a D_2_O-exchangeable singlet signal in the range of 5.51–5.61 ppm assigned to the amino group.

The reaction of compounds **5a,b** and **9a,b** with chloroacetyl chloride in the presence of potassium carbonate afforded compounds **6a,b** and **10a,b**, respectively [67,68]. The IR spectra of compounds **6a,b** and **10a,b** showed bands in the range of 3275–3271 cm^−1^ referring to the NH group along with a new absorption peak attributed to the acetamide carbonyl moiety at 1689 cm^−1^ for compound **6a**, 1693 cm^−1^ for compound **6b,** and 1701 cm^−1^ for both compounds **10a,b**. Furthermore, the ^1^H NMR spectra of compounds **6a,b** and **10a,b** confirmed the disappearance of the NH_2_ signal of compounds **5a,b** and **9a,b** along with the appearance of a D_2_O-exchangeable singlet signal assigned for acetamide NH at 10.4–10.5 ppm. It also showed a singlet signal at 4.7–4.73 ppm corresponding to CH_2_ protons of the acetamide moiety. Furthermore, the ^13^C NMR spectra of compounds **6a,b** and **10a,b** displayed a signal at 79.12 ppm assigned to the CH_2_ carbons of the acetamide group in addition to signals at 167.12 and 168.30 ppm attributed to the C=O carbon of the acetamide group and the C=O at C-4 of the quinazoline ring, respectively.

Compounds **7a**–**c** and **11a**–**c** were synthesized through the reaction of compounds **6a** and **10a**, respectively, with substituted phenols in the presence of potassium carbonate [67]. Similarly, heating of the substituted phenols and K_2_CO_3_ with compounds **6b** and **10b** afforded compounds **7d**–**f** and **11d**–**f**, respectively. 

We proved the structure of compounds **7a**–**f** and **11a**–**f** by various spectral data. The ^1^H NMR spectra of compounds **7a**–**f** and **11a**–**f** showed an increase in the aromatic protons at 6.56–8.59 ppm due to protons of the substituted phenolic ring. Furthermore, the ^1^H NMR spectrum of compounds **7a**, **7c**, **7d**, **7f**, **11a**, **11c**, **11d,** and **11f** showed a singlet signal at 2.24–2.30 corresponding to the CH_3_ protons of the substituted phenolic ring. The ^13^C NMR of compounds **7a**–**f** and **11a**–**f** represented an increase in the number of signals assigned for aromatic carbons at 107.64–168.78 ppm.

### 3.2. Biological Evaluation

#### 3.2.1. Screening of Compounds Using HCV GT 1b Replicon Assay

**Figure 3:**
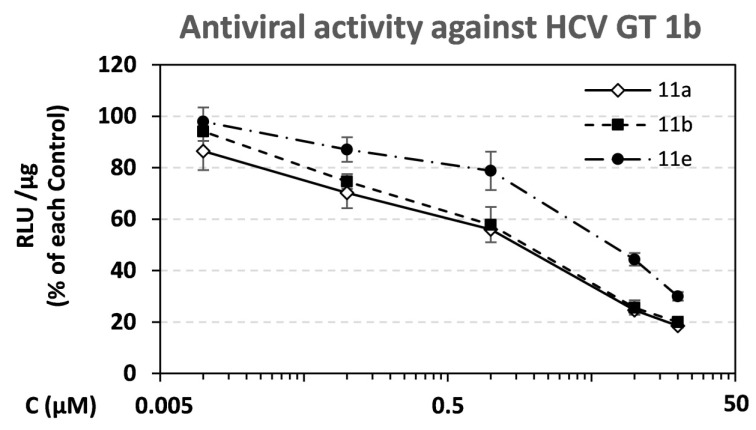
Dose–response curves for compounds **11a**, **11b,** and **11e** against replication of HCV GT 1b. Serial dilutions of compounds were used to treat Huh5-2 replicon cells for 72 h. F-Luc activity was measured and expressed as relative light units (RLUs) per μg of total protein. Values from compound-treated cells were expressed as percentage of the ones from solvent-treated DMSO (control) cells. Bars represent mean values from 3 independent triplicate experiments. Error bars represent standard deviation (SD).

The anti-HCV potency of the synthesized compounds **1a,b**, **3a,b**, **4a,b**, **5a,b**, **6a,b**, **8a, b**, **9a,b**, **10a,b**, **7a**–**f**, and **11a**–**f** in cell culture was evaluated in the Huh5.2 stable cell line harboring the HCV GT 1b subgenomic reporter replicon (strain Con1). In this cell-based assay, the amount of firefly luciferase (F-Luc) co-expressed by the replicon directly correlates with the level of HCV RNA replication. We used compound concentrations that were not cytotoxic based on the results from the alamarBlue reduction cell viability assay. The results were expressed as values of the median effective concentration (EC_50_), defined as the concentration of compound that reduced the viral replication-driven F-Luc activity by 50% (Table 2). The approved drug daclatasvir was used as a positive control for viral inhibition in the replicon system. According to the data, compound **11a** exhibited the highest anti-viral potency (EC_50_ = 0.98 µM). Slightly lower was the activity of compound **11b** (EC_50_ = 1.38 µM). Compounds **11e** and **5a** also showed anti-HCV potency at a low micromolar concentration (EC_50_ 6.24 and 9.84 µM, respectively). For the most potent analogues **11a**, **11b,** and **11e**, dose–response curve analysis is presented (Figure 3).

#### 3.2.2. Cytotoxicity and Selectivity of the Synthesized Compounds

Determination of the median cytotoxic concentration (CC_50_) of the synthesized analogues, which represent the compound concentration causing 50% cell death (Table 2), showed that only three compounds, **11d**, **11e,** and **11f,** showed cytotoxic activity with CC_50_ values of 22.7 µM, 36.6 µM, and 27.4 µM, respectively. However, the SI value of the potent analogue **11e** is acceptable (SI = 5.86). The rest of the tested compounds did not exhibit significant cytotoxicity, providing a good safety profile as anti-HCV candidates. Analogues with the highest activity, **11a** and **11b**, also had high selectivity indices (SI = CC_50_/EC_50_), 160.71 and 71.75, respectively.

#### 3.2.3. Compound Activity against HCV **3a** and **4a** Replicon Cells

The anti-HCV activity of the analogues was also tested against other genotypes. Specifically, we measured their effect against viral replication-driven F-Luc activity in Huh7.5-3a and Huh7.5-4a stable cell lines containing the HCV GT 3a (strain S52) and 4a (strain ED43) replicons, respectively. Compounds **11b** and **11e** showed activity against GT 3a, and **11e** was the most active with EC_50_ = 8.4 μM (Table 2). Nevertheless, they exhibited no activity against GT 2a (data not shown). Compounds **11a** [EC_50_ = 0.98 μΜ (GT1b)] and **11b** [EC_50_ = 1.38 μΜ (GT1b), 11.65 μΜ (GT3a)] exhibited the highest anti-HCV potency. Similarly, compounds **11d** and **11e** demonstrated good anti-HCV activity but, unfortunately, with cytotoxic activity [CC_50_ = 22.7μΜ (**11d**), 36.6 (**11e**)]. We believe that the presence of a mono-substituted phenoxy ring in the para-position together with methyl substitution at C-2 of the quinazoline ring is essential for this potent anti-HCV activity.

#### 3.2.4. Validation of Compound Activity with Additional Assays

**Figure 4:**
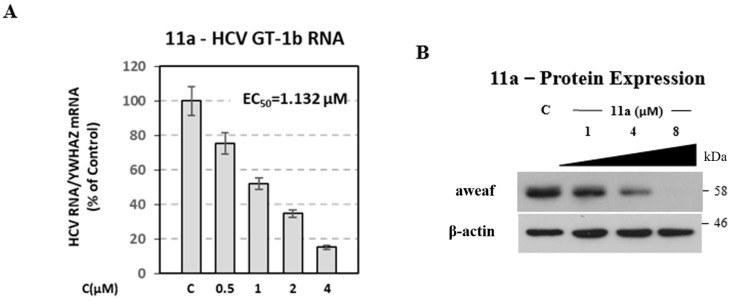
Inhibitory activity of analogue **11a** on HCV RNA and protein expression levels of the subgenomic 1b (Con1) replicon. Huh5-2 cells were treated with serial dilutions of **11a** or the solvent DMSO (control—C). (**A**) (+) strand HCV RNA was quantified by RT-qPCR. Values were normalized to the housekeeping gene 14-3-3-zeta polypeptide (YWHAZ) mRNA levels and expressed as percentage of those derived from cells treated with the solvent (control), which was set to 100%. Bars represent mean values obtained from three separate experiments in triplicate. Error bars represent standard deviation (SD). (**B**) Western blot analysis for the viral protein NS5A. β-actin was used as a loading control. A representative experiment of three independent replicates is shown.

The inhibition profile of the most promising compound **11a** measured by the luciferase assay in HCV GT 1b was confirmed by determining viral RNA and NS5A protein levels. Serial dilutions of the compound, or its solvent DMSO (control), were used to treat Huh5-2 replicon cells. Viral RNA was quantified by reverse transcription–quantitative polymerase chain reaction (RT-qPCR) and NS5A was evaluated by Western blotting. We found that compound **11a** caused a reduction in HCV RNA replication (Figure 4A), similar to the one in replication-derived luciferase activity with EC_50_ = 1.13 µM. Consistent results were obtained in the detection of HCV NS5A protein in Huh5-2 replicon cells, which was best visible at a low compound concentration (Figure 4B). 

#### 3.2.5. Combinatory Effect of Compound **11a** with DCV

**Figure 5:**
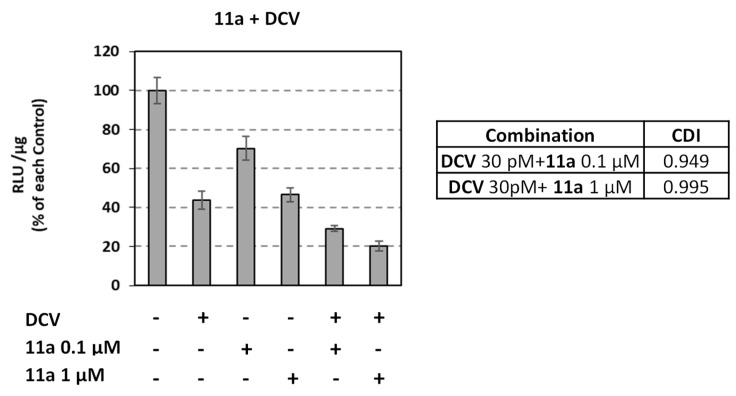
Combinatory activity of **11a** with DCV against HCV GT 1b (Con1). Huh5-2 cells were treated or not with the analogue in the presence or absence of 30 pM DCV. F-Luc activity was measured and expressed as RLU per μg of total protein. Values from compound-treated cells were expressed as percentage of those derived from cells treated with the solvent DMSO (control—C). CDI: Coefficient of drug interaction. Bars represent mean values obtained from three independent experiments in triplicate. Error bars represent standard deviation (SD).

To examine the activity of compound **11a** in combination with an approved NS5A inhibitor, the Huh5-2 replicon cell line was treated with **11a** in the presence or absence of daclatasvir (DCV) (Figure 5). The two compounds had an additive effect, as observed after determining the coefficient of the drug interaction (CDI ≈ 1).

### 3.3. Solubility Measurements

The observed water solubility of compound **11a** is **2.7** µgm/mL and for compound **11b** is **3.5** µgm/mL

### 3.4. In Silico Studies

**Figure 6:**
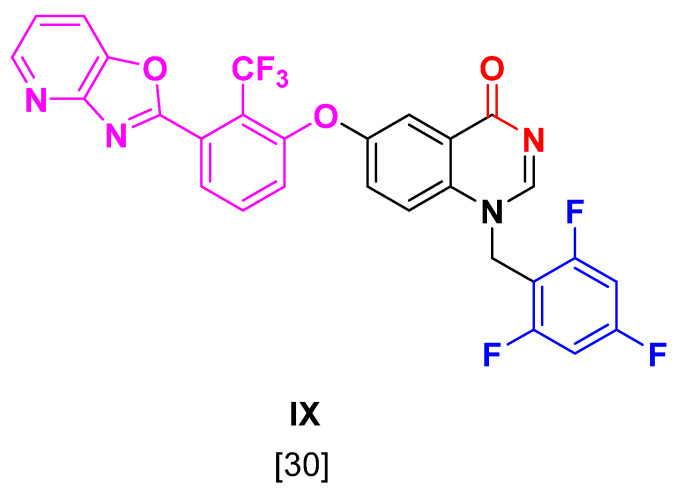
Structure of co-crystallized ligand of 4JU1 used for redocking.

Computational docking studies were carried out with Glide, Schrödinger platform [60,61,62] to investigate the binding mode of **11a**–**f** compounds. The crystal structure of HCV NS5B polymerase with Protein Data Bank code 4JU1 (resolution 2.90 Å, crystallization at pH 7.5) was employed [39], due to the similarity of the azabenzoxazole moiety in the bound ligand **IX** (Figure 6) with the motif of **11a**–**f**. The protein was set up for docking with Glide, Schrödinger [69,70,71] by removing all water molecules (except water molecule 3186, which is located in close proximity to the ligand and Leu497 and is also conserved to the position in PDB crystals of HCV NS5B polymerase 4JU2 and 4JTW) after superposition with 4JU1 and non-protein atoms, while keeping the magnesium ions. Optimization and minimization of the protein and protonation of the amino acids were performed using the Protein Preparation Wizard, Schrödinger [72] under standard conditions. The ligand structures were either built in Maestro, Schrödinger (compounds **11a**–**f**) or extracted from crystal structures (a co-crystallized ligand of 4JU1 used for redocking, a co-crystallized ligand of 4JU2 used for crossdocking—cf. Figure 1). Protonation and energy minimization of the ligand structures was conducted with LigPrep, Schrödinger under standard conditions with the default protocol. Both the redocking and crossdocking yielded top XP score poses with low RMS values of 0.5425 and 1.5999, respectively, compared to the original crystal structures. However, rigid docking with the same protocol failed to place compounds **11a**–**f** in the binding pocket and resulted in them drifting towards the left-hand side. This occurred due to the rigidity of the phenylquinazolinone core compared to benzylquinazolinone, with the former not having enough space to enter the cavity. The latter is not only more flexible but also the methylene bridge of the benzyl moiety allows the respective phenyl group to be positioned at an optimal angle to geometrically fit the cavity [39]. Nonetheless, this part of the pocket is comprised of flexible side groups of amino acids, such as Leu419, Met423, and Arg422, which could accommodate the phenylquinazolinone core in a less static simulation after minimal displacement. Thus, induced-fit docking was performed using the default protocol of Glide [73,74,75]. Indeed, the aforementioned amino acids move slightly (RMSD: Leu419 0.11–0.25; Met423 0.08–0.11; Arg422 1.02–1.12) and enlarge the existing cleft, therefore allowing the phenylquinazolinone scaffold to reside there. Furthermore, an MMGBSA free energy calculation of binding was conducted using Prime, Schrödinger [76,77] to the induced-fit docking output to facilitate comparison between the different poses. Selection of the top poses for each ligand was conducted based on the docking score, the free energy of binding (MMGBSA), and the RMSD value against the bound quinazolinone core in 4JU1, with the rationale that these structurally similar ligands should, in principle, exhibit a similar binding mode, with minimal spatial deviations of the key scaffold, which forms two critical hydrogen bonds with the enzyme. Again, redocking and crossdocking resulted in adequate ligand RMSD for induced-fit docking, at 1.23 and 1.62, respectively (compared to the original crystal structures), and with even lower ΔG_bind_ energies (cf. Table 3). 

**Figure 7:**
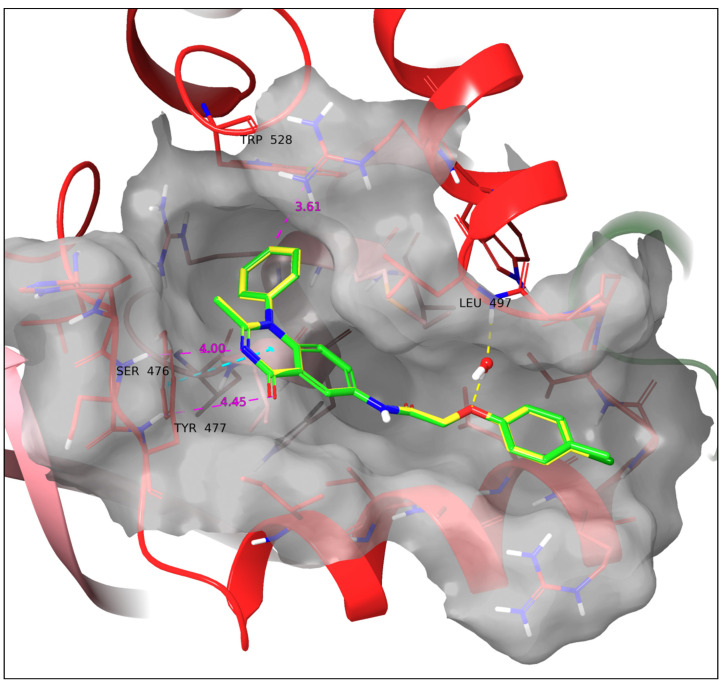
Docking poses of **11a** (green) and **11b** (yellow). The two ligands have an identical binding mode. A water bridge with Leu497 and a sandwich π–π stacking with Tyr477 are formed. Yellow dotted line: Hydrogen bond. Cyan dotted line: π–π interaction. All the above SARs were identified by employing the default settings of Maestro. Violet dotted line and number: Atom/ring distance in Å (measured manually).

The input from the crystal structures suggests that the most important SAR of the lead compounds **IX** and **VIII** applicable in our case are the two hydrogen bonds formed by the quinazolinone core and Ser476 and Tyr477. Ligands **11a** and **11b** share identical binding modes and SARs, which is in accordance with their high level of similarity. Although they do not form hydrogen bonds with Ser476 and Tyr477, the respective distances with the quinazolinone heteroatoms are 4 and 4.45 Å, which indicate that in a dynamic environment, their formation could be likely, as also depicted by their low RMSD values (cf. Table 3). However, the phenylquinazolinone scaffold forms a sandwich π–π stacking with the side group of Tyr477, which stabilizes the ligand in this favorable vicinity, rendering the aforementioned hydrogen bond formation more probable. In addition, a water bridge is formed between the ether O and the Leu497 backbone. The identical SARs of **11a** and **11b** could indicate that the slight difference they exhibit in biological activity might be the result of parameters other than their binding mode, e.g., their difference in solubility as described above (Figure 7). 

**Figure 8:**
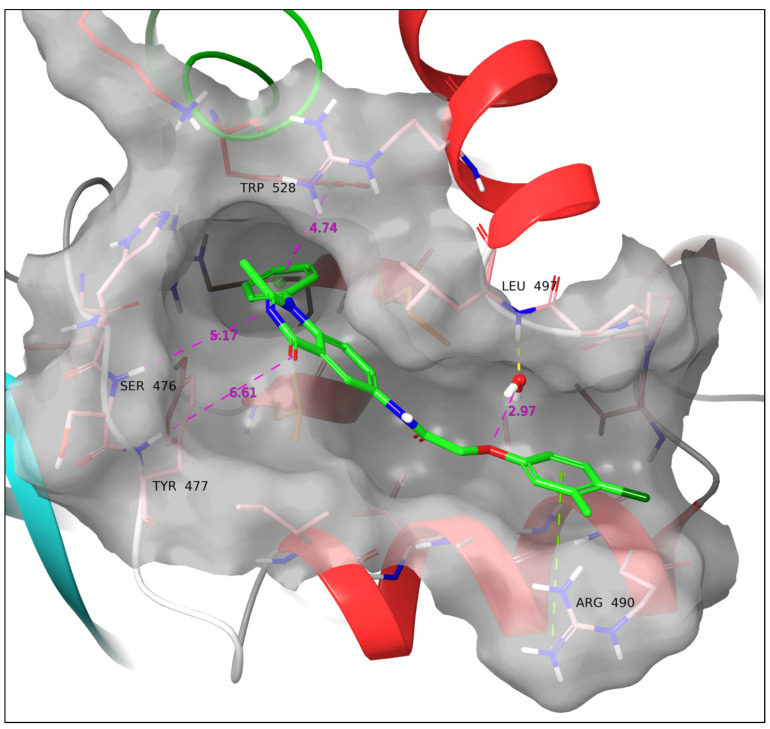
Docking pose of **11c**. Cation–π stacking is formed with Arg490, while the ligand drifts the furthest away from Ser476 and Tyr477. Yellow dotted line: Hydrogen bond. Green dotted line: Cation–π interaction. All the above SARs were identified by employing the default settings of Maestro. Violet dotted line and number: Atom/ring distance in Å (measured manually).

Ligand **11c** exhibits the longest distance between the phenylquinazolinone heteroatoms and Ser476 and Tyr477 (Ser476-aromatic N: 5.17 Å; Tyr477-carbonyl O: 6.61 Å). The reserved crystal water forms a hydrogen bond with Leu497 but not with the ligands’ ether O, mostly due to the discrepancy in the conserved orientation of the water hydrogen since the latter is in very close proximity to it (aqueous O-ether O: 2.97 Å). Hence, in a less static environment for the crystal water, the water bridge between **11c**-Leu497 is expected to take place. Moreover, a cation–π interaction between the protonated guanidine of Arg490 is observed (Figure 8).

**Figure 9:**
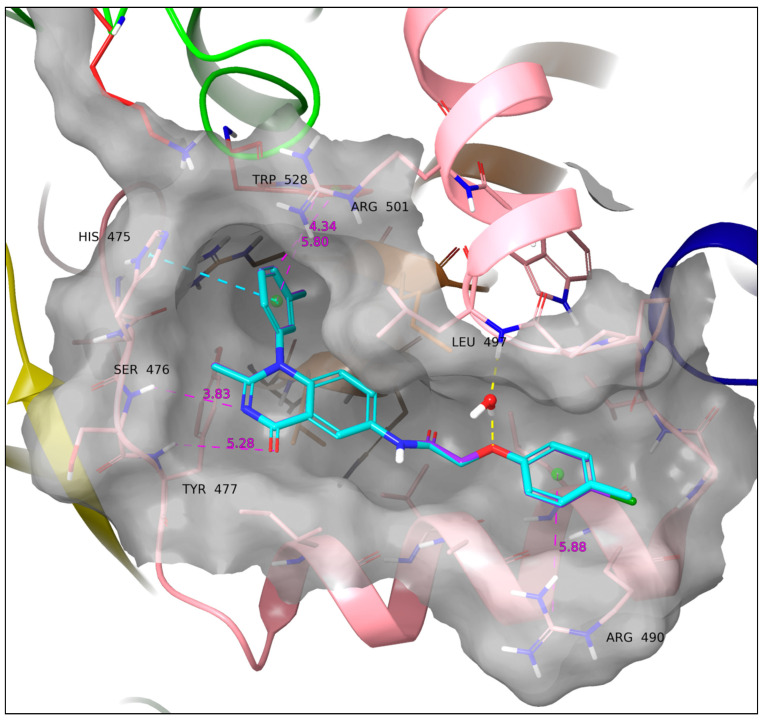
Docking poses of **11d** (cyan) and **11e** (purple). The two ligands have an identical binding mode. A water bridge with Leu497 and a sandwich π–π stacking with His475 are formed. Yellow dotted line: Hydrogen bond. Cyan dotted line: π–π interaction. All the above SARs were identified employing the default settings of Maestro. Violet dotted line and number: Atom/ring distance in Å (measured manually).

Compounds **11d** and **11e** also share very similar binding modes and SARs, again in accordance with their structural similarity; however, with certain differences compared to the binding mode of **11a** and **11b**. Ligands **11d** and **11e** are closer to Ser476 but, due to their orientation, drift further from Tyr477 than **11a** and **11b** (Ser476-aromatic N: 3.83 Å; Tyr477-carbonyl O: 5.28 Å) without forming hydrogen bonds. They both form a water bridge between the ether O and the Leu497 backbone and a sandwich π–π stacking with His475 (Figure 9). 

**Figure 10:**
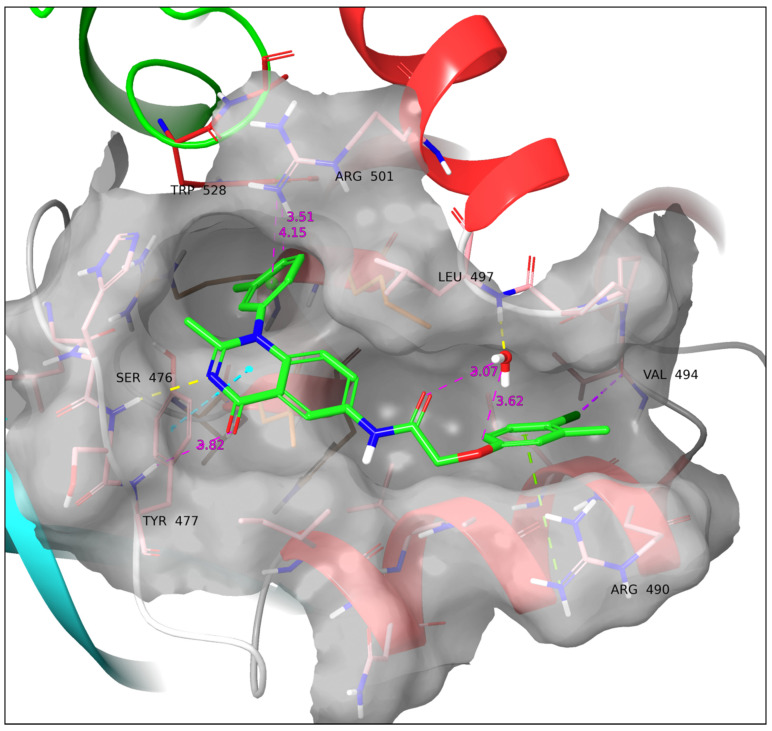
Docking pose of **11f**. A hydrogen bond with Ser476, a sandwich π–π stacking with Ty477, a cation–π interaction with Arg490, and a halogen bond with Val494 are formed. Yellow dotted line: Hydrogen bond. Cyan dotted line: π–π interaction. Green dotted line: cation–π interaction. Purple dotted line: Halogen bond. All the above SARs were identified employing the default settings of Maestro. Violet dotted line and number: Atom/ring distance in Å (measured manually).

Compound **11f** displays the richest SAR of all the series while retaining the lowest RMSD of its quinazolinone core compared to the co-crystalized ligand (cf. Table 3). It forms a cation–π interaction with Arg490, a sandwich π–π stacking with the side group of Tyr477, a hydrogen bond with the backbone of Ser476, and a halogen bond with the backbone of Val494. The hydrogen bond with Tyr477 is not formed, albeit the distance Tyr477-carbonyl O is 3.82 Å. The water bridge with Leu497 is not formed, similarly to **11c**; however, the crystal water molecule is close to the ether O (3.62 Å) and surprisingly even closer to the amide carbonyl O (3.07 Å) (Figure 10).

As observed in all docking poses, the phenyl group of the phenylquinazolinone core can be well accommodated within the newly revealed cleft, even with an m-CH_3_ substitution. In addition, its proximity to Trp528, His475, and the guanidine group of Arg501 open a possibility that the phenyl might form π–π interactions (His475, Trp528) and cation–π interactions (Arg501) in a dynamic environment. Furthermore, it is interesting that between the two pairs with identical binding modes, i.e., **11a**, **11b,** and **11d**, **11e,** the only difference is the exchange of a p-CH_3_ to a p-Cl, but this nonetheless results in differences in the respective EC_50_. We have shown that this structural characteristic also results in a difference in water solubility for **11a** and **11b.** Furthermore, since a cation–π interaction with the guanidine of Arg490 has been illustrated by our model for compounds **11c** and **11f,** which bear both groups, it cannot be excluded that such an interaction may take place during the binding process of the other ligands. In that case, the methyl substitution would enhance a possible cation–π interaction by enriching the electron density of the ring compared to the chloro-analogue—a feature difficult to simulate accurately in the MM environment.

**Figure 11:**
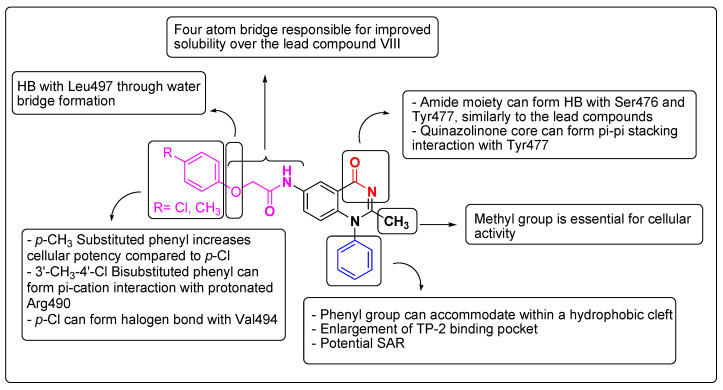
Plausible SAR of the target synthesized compounds **11a**–**f**.

As presented above, the series of **11a**–**f** displays a variety of SARs; however, not all of them are of equal importance (Figure 11). In addition to exhibiting optimal SAR, a formidable ligand should also render a promising binding energy profile based on prominent enthalpic interactions. Thus, the MMGBSA-calculated ΔG_bind_ ranks the interactions of each ligand and provides supplementary profiling, according to which (a) the co-crystallized ligands **VIII and IX** have the lowest ΔG_bind_; (b) the ligands **11a** and **11b** are next, although at a higher energy level; (c) the rest of the ligands follow at an even higher energy level. A trend can be clearly seen between the values of ΔG_bind_ and the EC_50_ values. This profiling highlights that the formation of the two hydrogen bonds between Ser476 and Tyr477 and the quinazolinone core, or at least the optimal positioning of the latter towards the critical amino acids via other interactions (e.g., π-π stacking in **11a** and **11b**), is of the highest importance, similarly to the lead compound binding mode. Ligands lacking these interactions and drifting away from this vicinity resulted in higher ΔG_bind_ and EC_50_ values. Furthermore, it is notable that compounds bearing a 3-methyl group on the quinazolinone phenyl groups receive an energy penalty due to rotation hindrance and clash with the protein cleft.

### 3.5. Lipophilicity and Solubility Parameters Calculations

The ClogP and Log S values for all compounds were illustrated in Table 4. The target compounds **7a**–**f** and **11a**–**f** have lower LogP values (3.52–4.79) than the reported lead compound **VII** (LogP = 8.09) and the ligand compounds **VIII** and **IX** (LogP = 9.15 and 9.29, respectively). Moreover, the target compounds **7a**–**f** and **11a**–**f** have better LogS values (−5.67 ~ −4.72) than compound **VII** (Log S = −6.02) and the ligand compounds **VIII** and **IX** (Log S = −6.84 and −7.22, respectively). 

## 4. Conclusions

In conclusion, a novel series of anthranilic acid, 1-substituted phenyl-4(1*H*)-quinazolinone, and 2-methyl-1-substituted phenyl-4(1*H*)-quinazolinone derivatives have been prepared and tested for their antiviral potency against various HCV GTs. Compound **11a** was the most potent against HCV GT 1b (EC_50_ = 0.98 µM) and showed a synergistic effect in combination with an approved NS5A inhibitor daclatasvir (DCV). The SAR study of the synthesized compounds revealed that the presence of both the mono-substituted phenoxy ring in the *para*-position and the methyl group at C-2 of the quinazolinone ring, as seen in compounds **11a**, **11b**, **11d,** and **11e**, is essential for potent antiviral activity. Due to the proximity of the C-2 methyl group to the phenyl group of the quinazolinone scaffold, it could be hypothesized that its presence may facilitate the opening of the cleft, rendering it better approachable by the rigid phenyl ring. Our computational investigations show that the phenyl analogues, which by a rigid approach were deemed not to fit in the binding site, can reach a lipophilic cleft within the pocket where they can be accommodated. As exhibited in the docking poses of **11a**–**f**, there are possible SARs that might take place between the phenyl group and the newly revealed cleft in a dynamic environment. However, as shown both in the computational modelling and the pharmacologic data, these prospective SARs would be of less significance than the ones of the benzyl analogues, since neither our model showed any clear solid interactions forming in this cleft nor did our pharmacological evaluation result in higher affinity than the lead compounds. Nonetheless, it is important to exhibit that by minimal movement of lipophilic side chains, the known binding pocket can be enlarged to accommodate more rigid ligands, but also that this new cleft presents opportunities for SAR, which can be exploited in future drug design. Furthermore, the hydrogen bonds from the quinazolinone core were highlighted as the most important SARs. These findings may play an important role in further structure optimization aimed at the development and production of more potent anti-HCV agents.

## Figures and Tables

**Table 1:** Reported EC_50_ and calculated lipophilic and solubility parameters for compounds **I**–**VIII**.

Compound	EC_50_ (μΜ)	ClogP ^a^	Log S ^b^
**I**	-	6.67	−5.77
**II**	0.026 ^c^	5.49	−5.88
**III**	-	2.97	−4.35
**IV**	-	5.68	−5.68
**V**	>30 ^d^	5.18	−5.10
**VI**	0.095 ^d^	7.31	−6.00
**VII**	0.38 ^d^	8.09	−6.02
**VIII**	0.31 ^d^	9.15	−6.84

^a^ Calculated by online Swiss ADME website application [45,46]. ^b^ ESOL calculated by online Swiss ADME website application [45,46]. Solubility class: Log S scale (insoluble < −10 < poorly soluble < −6 < moderately soluble < −4 < soluble < −2 < very soluble < 0 < highly soluble) [47]. ^c^ HCV GT-1b replicon containing Huh-7 cells [36]. ^d^ HCV GT-1b replicon luciferase reporter assay (n ≥ 2) [38].

**Table 2:** EC_50_, CC_50_, and SI values of compounds in HCV replicon cell lines of GT 1b (Con1), GT 3a (S52), and GT 4a (ED43).

	HCV Replicon 1b			HCV Replicon 3a		HCV Replicon 4a	
Code	CC_50_ (μΜ)	EC_50_ (μΜ)	SI	EC_50_ (μΜ)	SI	EC_50_ (μΜ)	SI
**1a**	>200	>10	-	>10	-	>10	-
**1b**	>200	>10	-	>10	-	>10	-
**3a**	70.69 ± 0.04	>10	-	>10	-	>10	-
**3b**	161.3 ± 0.07	>10	-	>10	-	>10	-
**4a**	>200	>10	-	>10	-	>10	-
**4b**	64.9 ± 0.03	>10	-	>10	-	>10	-
**5a**	>200	9.84 ± 0.57	>20.32	>10	-	>10	-
**5b**	>200	>10	-	>10	-	>10	-
**6a**	104.2 ± 0.03	>10	-	>10	-	>10	-
**6b**	143.3 ± 14.24	>10	-	>10	-	>10	-
**8a**	>200	>10	-	>10	-	>10	-
**8b**	151.1 ± 5.41	>10	-	>10	-	>10	-
**9a**	>200	>10	-	>10	-	>10	-
**9b**	>200	>10	-	>10	-	>10	-
**10a**	87.47 ± 0.24	>10	-	>10	-	>10	-
**10b**	177.2 ± 0.03	>10	-	>10	-	>10	-
**7a**	84.67 ± 13.96	>10	-	>10	-	>10	-
**7b**	154.3 ± 3.71	>10	-	>10	-	>10	-
**7c**	75.22 ± 9.24	>10	-	>10	-	>10	-
**7d**	50.13 ± 1.77	>10	-	>10	-	>10	-
**7e**	47.6 ± 5.56	>10	-	>10	-	>10	-
**7f**	105.4 ± 10.30	>10	-	>10	-	>10	-
**11a**	157.5 ± 0.03	0.98 ± 0.06	160.71	>10	-	>10	-
**11b**	99.01 ± 0.09	1.38 ± 0.04	71.75	11.65 ± 1.26	8.5	>10	-
**11c**	>200	>10	-	>10	-	>10	-
**11d**	22.7 ± 2.23	>1	-	>10	-	>10	-
**11e**	36.6 ± 4.19	6.24 ± 0.04	5.86	8.4 ± 0.15	4.36	15.21 ± 0.29	2.4
**11f**	27.4 ± 2.71	>1	-	>10	-	>10	-
**Daclatasvir**	17.7	26.6 × 10^−6^ ± 4.6 × 10^−6^	680,769.23	21.2 × 10^−6^ ± 3.1 × 10^−7^	2120.27	0.008 ± 0.001	835,299.67

**Table 3:** Docking data.

Compound	Docking	RMSD	MMGBSA ΔG_bind_ (kcal/mol)	Glide XP Score	IFD Score
**IX (redock)**	Rigid	0.5425 ^a^	−88.83	−10.098	-
**VIII (crossdock)**	Rigid	1.5999 ^b^	−82.11	−9.296	-
**IX (redock)**	Induced fit	1.2336 ^a^	−92.19	−10.461	−1171.83
**VIII (crossdock)**	Induced fit	1.6153 ^b^	−92.37	−10.126	−1171.50
**11a**	Induced fit	2.6138 ^c^	−69.86	−6.349	−1167.50
**11b**	Induced fit	2.6174 ^c^	−71.65	−7.311	−1168.47
**11c**	Induced fit	2.5848 ^c^	−60.81	−5.414	−1167.01
**11d**	Induced fit	2.2737 ^c^	−59.60	−6.497	−1167.61
**11e**	Induced fit	2.2734 ^c^	−61.18	−5.765	−1166.87
**11f**	Induced fit	1.2665 ^c^	−61.90	−5.091	−1166.04

^a^ Calculated versus the co-crystalized **IX** ligand in 4JU1. ^b^ Calculated versus the co-crystallized **VIII** ligand in 4JU2. ^c^ Calculated versus the quinazolinone core of the co-crystallized **IX** ligand in 4JU1.

**Table 4:** Calculated lipophilic and solubility parameters for the synthesized compounds and the ligand compound IX.

Compounds	ClogP ^a^	Log S ^b^	Compounds	ClogP	Log S	Compounds	ClogP	Log S
**1a**	3.04	−4.52	7b	3.87	−5.01	11a	3.83	−4.78
**1b**	3.35	−4.68	7c	4.17	−5.31	11b	4.17	−5.07
**3a**	3.22	−4.57	7d	3.83	−5.01	11c	4.48	−5.37
**3b**	3.53	−4.73	7e	4.17	−5.31	11d	4.14	−5.08
**4a**	2.29	−3.39	7f	4.48	−5.61	11e	4.48	−5.37
**4b**	2.6	−3.68	8a	2.6	−3.44	11f	4.79	−5.67
**5a**	1.98	−3.02	8b	2.91	−3.73	IX	9.29	−7.22
**5b**	2.28	−3.30	9a	2.28	−3.06			
**6a**	2.37	−3.47	9b	2.59	−3.35			
**6b**	2.68	−3.77	10a	2.68	−3.53			
**7a**	3.52	−4.72	10b	2.99	−3.83			

^a^ Calculated by online Swiss ADME website application [45,46]. ^b^ ESOL calculated by online Swiss ADME website application [45,46]. Solubility class: log S scale (insoluble < −10 < poorly soluble < −6 < moderately soluble < −4 < soluble < −2 < very soluble < 0 < highly soluble) [47].

## Data Availability

All relevant data are within the manuscript.

## References

[1] Cohen J. (1999). The Scientific Challenge of Hepatitis C. Science.

[2] Lee W.M. (1990). Hepatitis C. Curr. Opin. Infect. Dis..

[3] Pawlotsky J.-M., Negro F., Aghemo A., Berenguer M., Dalgard O., Dusheiko G., Marra F., Puoti M., Wedemeyer H. (2020). EASL Recommendations on Treatment of Hepatitis C: Final Update of the Series. J. Hepatol..

[4] Rabaan A.A., Al-Ahmed S.H., Bazzi A.M., Alfouzan W.A., Alsuliman S.A., Aldrazi F.A., Haque S. (2020). Overview of Hepatitis C Infection, Molecular Biology, and New Treatment. J. Infect. Public Health.

[5] WHO Publishes Updated Guidance on Hepatitis C Infection–with New Recommendations on Treatment of Adolescents and Children, Simplified Service Delivery and Diagnostics. https://www.who.int/news/item/24-06-2022-WHO-publishes-updated-guidance-on-hepatitis-C-infection.

[6] Feld J.J., Hoofnagle J.H. (2005). Mechanism of Action of Interferon and Ribavirin in Treatment of Hepatitis C. Nature.

[7] Marks K.M., Jacobson I.M. (2012). The First Wave: HCV NS3 Protease Inhibitors Telaprevir and Boceprevir. Antivir. Ther..

[8] Cross T.J.S., Antoniades C.G., Harrison P.M. (2008). Current and Future Management of Chronic Hepatitis C Infection. Postgrad. Med. J..

[9] Paula T., Pablo R., Eugenia V., Pablo B., Sabino P., Jose M., Antonio M., Dolores H., Pablo L., Javier G.-S. (2009). New Drug Targets for Hepatitis C and Other Flaviviridae Viruses. Infect. Disord.-Drug Targets.

[10] Pockros P.J. (2012). Interferon-Free Hepatitis C Therapy. Drugs.

[11] Lange C.M., Zeuzem S. (2013). Perspectives and Challenges of Interferon-Free Therapy for Chronic Hepatitis C. J. Hepatol..

[12] Asselah T., Marcellin P., Schinazi R.F. (2018). Treatment of Hepatitis C Virus Infection with Direct-Acting Antiviral Agents: 100% Cure?. Liver Int..

[13] Alarfaj S.J., Alzahrani A., Alotaibi A., Almutairi M., Hakami M., Alhomaid N., Alharthi N., Korayem G.B., Alghamdi A. (2022). The Effectiveness and Safety of Direct-Acting Antivirals for Hepatitis C Virus Treatment: A Single-Center Experience in Saudi Arabia. Saudi Pharm. J..

[14] Yee J., Carson J.M., Hajarizadeh B., Hanson J., O’Beirne J., Iser D., Read P., Balcomb A., Doyle J.S., Davies J. (2022). High Effectiveness of Broad Access Direct-Acting Antiviral Therapy for Hepatitis C in an Australian Real-World Cohort: The REACH-C Study. Hepatol. Commun..

[15] Sencanski M., Glisic S. (2020). Direct-Acting Antiviral Drugs for Treatment of Hepatitis C Virus Infection-Clinical Trials Data and Chemistry of NS3/4a Protease Inhibitors. Frontiers in Clinical Drug Research: Anti-Infectives.

[16] Tagkou N.M., Goossens N., Negro F. (2022). Impact of Direct-Acting Antivirals on the Recurrence of Hepatocellular Carcinoma in Chronic Hepatitis C. Hepatoma Res..

[17] Dietz C., Maasoumy B. (2022). Direct-Acting Antiviral Agents for Hepatitis C Virus Infection—From Drug Discovery to Successful Implementation in Clinical Practice. Viruses.

[18] Bartenschlager R., Lohmann V., Penin F. (2013). The Molecular and Structural Basis of Advanced Antiviral Therapy for Hepatitis C Virus Infection. Nat. Rev. Microbiol..

[19] Moradpour D., Penin F., Rice C.M. (2007). Replication of Hepatitis C Virus. Nat. Rev. Microbiol..

[20] Kish T., Aziz A., Sorio M. (2017). Hepatitis C in a New Era: A Review of Current Therapies. Pharm. Ther..

[21] Behrens S.E., Tomei L., De Francesco R. (1996). Identification and Properties of the RNA-Dependent RNA Polymerase of Hepatitis C Virus. EMBO J..

[22] Moradpour D., Brass V., Bieck E., Friebe P., Gosert R., Blum H.E., Bartenschlager R., Penin F., Lohmann V. (2004). Membrane Association of the RNA-Dependent RNA Polymerase Is Essential for Hepatitis C Virus RNA Replication. J. Virol..

[23] Huang Z., Murray M.G., Secrist J.A. (2006). Recent Development of Therapeutics for Chronic HCV Infection. Antivir. Res..

[24] Ago H., Adachi T., Yoshida A., Yamamoto M., Habuka N., Yatsunami K., Miyano M. (1999). Crystal Structure of the RNA-Dependent RNA Polymerase of Hepatitis C Virus. Structure.

[25] Bressanelli S., Tomei L., Roussel A., Incitti I., Vitale R.L., Mathieu M., De Francesco R., Rey F.A. (1999). Crystal Structure of the RNA-Dependent RNA Polymerase of Hepatitis C Virus. Proc. Natl. Acad. Sci. USA.

[26] Weber P.C., Lesburg C.A., Cable M.B., Ferrari E., Hong Z., Mannarino A.F. (1999). Crystal Structure of the RNA-Dependent RNA Polymerase from Hepatitis C Virus Reveals a Fully Encircled Active Site. Nat. Struct. Biol..

[27] Bressanelli S., Tomei L., Rey F.A., De Francesco R. (2002). Structural Analysis of the Hepatitis C Virus RNA Polymerase in Complex with Ribonucleotides. J. Virol..

[28] Barreca M.L., Iraci N., Manfroni G., Cecchetti V. (2011). Allosteric Inhibition of the Hepatitis C Virus NS5B Polymerase: In Silico Strategies for Drug Discovery and Development. Future Med. Chem..

[29] Horsley-Silva J.L., Vargas H.E. (2017). New Therapies for Hepatitis C Virus Infection. Gastroenterol. Hepatol..

[30] Zhou Z., Zhang J., Zhou E., Ren C., Wang J., Wang Y. (2022). Small Molecule NS5B RdRp Non-Nucleoside Inhibitors for the Treatment of HCV Infection: A Medicinal Chemistry Perspective. Eur. J. Med. Chem..

[31] Love R.A., Parge H.E., Yu X., Hickey M.J., Diehl W., Gao J., Wriggers H., Ekker A., Wang L., Thomson J.A. (2003). Crystallographic Identification of a Noncompetitive Inhibitor Binding Site on the Hepatitis C Virus NS5B RNA Polymerase Enzyme. J. Virol..

[32] Yan S., Appleby T., Larson G., Wu J.Z., Hamatake R.K., Hong Z., Yao N. (2007). Thiazolone-Acylsulfonamides as Novel HCV NS5B Polymerase Allosteric Inhibitors: Convergence of Structure-Based Drug Design and X-Ray Crystallographic Study. Bioorg. Med. Chem. Lett..

[33] Wang M., Ng K.K.S., Cherney M.M., Chan L., Yannopoulos C.G., Bedard J., Morin N., Nguyen-Ba N., Alaoui-Ismaili M.H., Bethell R.C. (2003). Non-Nucleoside Analogue Inhibitors Bind to an Allosteric Site on HCV NS5B Polymerase. J. Biol. Chem..

[34] Beaulieu P.L., Coulombe R., Duan J., Fazal G., Godbout C., Hucke O., Jakalian A., Joly M.-A., Lepage O., Llinàs-Brunet M. (2013). Structure-Based Design of Novel HCV NS5B Thumb Pocket 2 Allosteric Inhibitors with Submicromolar Gt1 Replicon Potency: Discovery of a Quinazolinone Chemotype. Bioorg. Med. Chem. Lett..

[35] Malancona S., Donghi M., Ferrara M., Martin Hernando J.I., Pompei M., Pesci S., Ontoria J.M., Koch U., Rowley M., Summa V. (2010). Allosteric Inhibitors of Hepatitis C Virus NS5B Polymerase Thumb Domain Site II: Structure-Based Design and Synthesis of New Templates. Bioorg. Med. Chem..

[36] Canales E., Carlson J.S., Appleby T., Fenaux M., Lee J., Tian Y., Tirunagari N., Wong M., Watkins W.J. (2012). Tri-Substituted Acylhydrazines as Tertiary Amide Bioisosteres: HCV NS5B Polymerase Inhibitors. Bioorg. Med. Chem. Lett..

[37] Stammers T.A., Coulombe R., Rancourt J., Thavonekham B., Fazal G., Goulet S., Jakalian A., Wernic D., Tsantrizos Y., Poupart M.-A. (2013). Discovery of a Novel Series of Non-Nucleoside Thumb Pocket 2 HCV NS5B Polymerase Inhibitors. Bioorg. Med. Chem. Lett..

[38] Stammers T.A., Coulombe R., Duplessis M., Fazal G., Gagnon A., Garneau M., Goulet S., Jakalian A., LaPlante S., Rancourt J. (2013). Anthranilic Acid-Based Thumb Pocket 2 HCV NS5B Polymerase Inhibitors with Sub-Micromolar Potency in the Cell-Based Replicon Assay. Bioorg. Med. Chem. Lett..

[39] Hucke O., Coulombe R., Bonneau P., Bertrand-Laperle M., Brochu C., Gillard J., Joly M.-A., Landry S., Lepage O., Llinàs-Brunet M. (2014). Molecular Dynamics Simulations and Structure-Based Rational Design Lead to Allosteric HCV NS5B Polymerase Thumb Pocket 2 Inhibitor with Picomolar Cellular Replicon Potency. J. Med. Chem..

[40] Yang H., Hendricks R.T., Arora N., Nitzan D., Yee C., Lucas M.C., Yang Y., Fung A., Rajyaguru S., Harris S.F. (2010). Cyclic Amide Bioisosterism: Strategic Application to the Design and Synthesis of HCV NS5B Polymerase Inhibitors. Bioorg. Med. Chem. Lett..

[41] Yan S., Larson G., Wu J.Z., Appleby T., Ding Y., Hamatake R., Hong Z., Yao N. (2007). Novel Thiazolones as HCV NS5B Polymerase Allosteric Inhibitors: Further Designs, SAR, and X-Ray Complex Structure. Bioorg. Med. Chem. Lett..

[42] Biswal B.K., Wang M., Cherney M.M., Chan L., Yannopoulos C.G., Bilimoria D., Bedard J., James M.N.G. (2006). Non-Nucleoside Inhibitors Binding to Hepatitis C Virus NS5B Polymerase Reveal a Novel Mechanism of Inhibition. J. Mol. Biol..

[43] Eltahla A., Luciani F., White P., Lloyd A., Bull R. (2015). Inhibitors of the Hepatitis C Virus Polymerase; Mode of Action and Resistance. Viruses.

[44] Hong Z., Cameron C.E., Walker M.P., Castro C., Yao N., Lau J.Y.N., Zhong W. (2001). A Novel Mechanism to Ensure Terminal Initiation by Hepatitis C Virus NS5B Polymerase. Virology.

[45] Wildman S.A., Crippen G.M. (1999). Prediction of Physicochemical Parameters by Atomic Contributions. J. Chem. Inf. Comput. Sci..

[46] Daina A., Michielin O., Zoete V. (2017). SwissADME: A Free Web Tool to Evaluate Pharmacokinetics, Drug-Likeness and Medicinal Chemistry Friendliness of Small Molecules. Sci. Rep..

[47] Delaney J.S. (2004). ESOL: Estimating Aqueous Solubility Directly from Molecular Structure. J. Chem. Inf. Comput. Sci..

[48] Baqi Y., Müller C.E. (2007). Catalyst-Free Microwave-Assisted Amination of 2-Chloro-5-Nitrobenzoic Acid. J. Org. Chem..

[49] Lan C., Xia Z.-N., Li Z.-H., Liang R.-H. (2012). Metal Catalyst-Free Amination of 2-Chloro-5-Nitrobenzoic Acid in Superheated Water. J. Chem. Res..

[50] Day M., Peters A.T. (1967). The Synthesis and Ultraviolet Spectra of Nitrodiphenylamine Disperse Dyes II—The Synthesis of Some Substituted 2- and 4-Nitrodiphenylamines. J. Soc. Dye. Colour..

[51] Kosáry J., Szabó I.K., Kasztreiner E. (1982). Synthesis and Biological Activity of 2,5-Diaminobenzoic Acids Stimulating the Biosynthesis of Prostaglandins. Pharmazie.

[52] Vrolijk J.M., Kaul A., Hansen B.E., Lohmann V., Haagmans B.L., Schalm S.W., Bartenschlager R. (2003). A Replicon-Based Bioassay for the Measurement of Interferons in Patients with Chronic Hepatitis C. J. Virol. Methods.

[53] Giannakopoulou E., Pardali V., Frakolaki E., Siozos V., Myrianthopoulos V., Mikros E., Taylor M.C., Kelly J.M., Vassilaki N., Zoidis G. (2019). Scaffold Hybridization Strategy towards Potent Hydroxamate-Based Inhibitors of Flaviviridae Viruses and Trypanosoma Species. Medchemcomm.

[54] Saeed M., Scheel T.K.H., Gottwein J.M., Marukian S., Dustin L.B., Bukh J., Rice C.M. (2012). Efficient Replication of Genotype 3a and 4a Hepatitis C Virus Replicons in Human Hepatoma Cells. Antimicrob. Agents Chemother..

[55] Dufner-Beattie J., O’Guin A., O’Guin S., Briley A., Wang B., Balsarotti J., Roth R., Starkey G., Slomczynska U., Noueiry A. (2014). Identification of AP80978, a Novel Small-Molecule Inhibitor of Hepatitis C Virus Replication That Targets NS4B. Antimicrob. Agents Chemother..

[56] Vassilaki N., Boleti H., Mavromara P. (2007). Expression Studies of the Core+1 Protein of the Hepatitis C Virus 1a in Mammalian Cells. FEBS J..

[57] Greenwood J.R., Calkins D., Sullivan A.P., Shelley J.C. (2010). Towards the Comprehensive, Rapid, and Accurate Prediction of the Favorable Tautomeric States of Drug-like Molecules in Aqueous Solution. J. Comput. Aided. Mol. Des..

[58] Shelley J.C., Cholleti A., Frye L.L., Greenwood J.R., Timlin M.R., Uchimaya M. (2007). Epik: A Software Program for PK a Prediction and Protonation State Generation for Drug-like Molecules. J. Comput. Aided. Mol. Des..

[59] Buckle D.R., Smith H. (1971). An Improved Synthesis of Substituted Benzoyl Acetates. J. Chem. Soc. C Org..

[60] Mistry R.N., Desai K.R. (2005). Studies on Synthesis of Some Novel Heterocyclic Azlactone Derivatives and Imidazolinone Derivatives and Their Antimicrobial Activity. E-Journal Chem..

[61] 2017 Reaction of Cinnamic Acid Chloride with Ammonia to Cinnamic Acid Amide. https://www.oc-praktikum.de/nop/en/instructions/pdf/2017_en.pdf.

[62] Rad-Moghadam K., Khajavi M.S. (1998). One-Pot Synthesis of Substituted Quinazolin-4(3H)-Ones under Microwave Irradiation. J. Chem. Res..

[63] Wang Y., Zhang M., Cao S., Lin H., Gao M., Li Z. (2012). Synthesis of 1-Substituted 4(1 H)-Quinazolinones Under Solvent-Free Conditions. Synth. Commun..

[64] Cao S.-L., Zhang M., Feng Y.-P., Jiang Y.-Y., Zhang N. (2008). Synthesis of 3-Aryl-4(3 H)-Quinazolinones from Anthranilic Acids and Triethyl Orthoformate. Synth. Commun..

[65] Srinivasa Reddy B., Naidu A., Dubey P.K. (2013). PEG-600-Mediated, Green and Efficient, Tandem Syntheses of N-Subtituted-2-Styrylquinazolin-4-Ones. Green Chem. Lett. Rev..

[66] Bellamy F.D., Ou K. (1984). Selective Reduction of Aromatic Nitro Compounds with Stannous Chloride in Non Acidic and Non Aqueous Medium. Tetrahedron Lett..

[67] Xia S., Wang L.Y., Sun H.Z., Yue H., Wang X.H., Tan J.L., Wang Y., Hou D., He X.Y., Mun K.C. (2013). Synthesis of N-Azaaryl Anilines: An Efficient Protocol via Smiles Rearrangement. Bull. Korean Chem. Soc..

[68] Praveen Kumar Darsi S.S., Nagamani K.S., Rama Devi B., Naidu A., Dubey P.K. (2011). Studies on N-Acetylation of Anilines with Acetyl Chloride Using Phase Transfer Catalysts in Different Solvents. Der Pharma Chem..

[69] Friesner R.A., Murphy R.B., Repasky M.P., Frye L.L., Greenwood J.R., Halgren T.A., Sanschagrin P.C., Mainz D.T. (2006). Extra Precision Glide: Docking and Scoring Incorporating a Model of Hydrophobic Enclosure for Protein−Ligand Complexes. J. Med. Chem..

[70] Halgren T.A., Murphy R.B., Friesner R.A., Beard H.S., Frye L.L., Pollard W.T., Banks J.L. (2004). Glide: A New Approach for Rapid, Accurate Docking and Scoring. 2. Enrichment Factors in Database Screening. J. Med. Chem..

[71] Friesner R.A., Banks J.L., Murphy R.B., Halgren T.A., Klicic J.J., Mainz D.T., Repasky M.P., Knoll E.H., Shelley M., Perry J.K. (2004). Glide: A New Approach for Rapid, Accurate Docking and Scoring. 1. Method and Assessment of Docking Accuracy. J. Med. Chem..

[72] Madhavi Sastry G., Adzhigirey M., Day T., Annabhimoju R., Sherman W. (2013). Protein and Ligand Preparation: Parameters, Protocols, and Influence on Virtual Screening Enrichments. J. Comput. Aided. Mol. Des..

[73] Farid R., Day T., Friesner R.A., Pearlstein R.A. (2006). New Insights about HERG Blockade Obtained from Protein Modeling, Potential Energy Mapping, and Docking Studies. Bioorg. Med. Chem..

[74] Sherman W., Beard H.S., Farid R. (2006). Use of an Induced Fit Receptor Structure in Virtual Screening. Chem. Biol. Drug Des..

[75] Sherman W., Day T., Jacobson M.P., Friesner R.A., Farid R. (2006). Novel Procedure for Modeling Ligand/Receptor Induced Fit Effects. J. Med. Chem..

[76] Jacobson M.P., Pincus D.L., Rapp C.S., Day T.J.F., Honig B., Shaw D.E., Friesner R.A. (2004). A Hierarchical Approach to All-Atom Protein Loop Prediction. Proteins Struct. Funct. Bioinforma..

[77] Jacobson M.P., Friesner R.A., Xiang Z., Honig B. (2002). On the Role of the Crystal Environment in Determining Protein Side-Chain Conformations. J. Mol. Biol..

